# From e-voucher to genomic data: Preserving archive specimens as demonstrated with medically important mosquitoes (Diptera: Culicidae) and kissing bugs (Hemiptera: Reduviidae)

**DOI:** 10.1371/journal.pone.0247068

**Published:** 2021-02-25

**Authors:** Silvia Andrade Justi, John Soghigian, David B. Pecor, Laura Caicedo-Quiroga, Wiriya Rutvisuttinunt, Tao Li, Lori Stevens, Patricia L. Dorn, Brian Wiegmann, Yvonne-Marie Linton

**Affiliations:** 1 Walter Reed Biosystematics Unit, Smithsonian Institution Museum Support Center, Suitland, MD, United States of America; 2 Entomology Branch, Walter Reed Army Institute of Research, Silver Spring, MD, United States of America; 3 Department of Entomology, Smithsonian Institution National Museum of Natural History, Washington, DC, United States of America; 4 Department of Entomology, North Carolina State University, Raleigh, NC, United States of America; 5 Viral Diseases Branch, Walter Reed Army Institute of Research, Silver Spring, MD, United States of America; 6 Department of Biology, University of Vermont, Burlington, VT, United States of America; 7 Department of Biological Sciences, Loyola University New Orleans, New Orleans, LA, United States of America; Universidade Federal do Rio de Janeiro, BRAZIL

## Abstract

Scientific collections such as the U.S. National Museum (USNM) are critical to filling knowledge gaps in molecular systematics studies. The global taxonomic impediment has resulted in a reduction of expert taxonomists generating new collections of rare or understudied taxa and these large historic collections may be the only reliable source of material for some taxa. Integrated systematics studies using both morphological examinations and DNA sequencing are often required for resolving many taxonomic issues but as DNA methods often require partial or complete destruction of a sample, there are many factors to consider before implementing destructive sampling of specimens within scientific collections. We present a methodology for the use of archive specimens that includes two crucial phases: 1) thoroughly documenting specimens destined for destructive sampling—a process called electronic vouchering, and 2) the pipeline used for whole genome sequencing of archived specimens, from extraction of genomic DNA to assembly of putative genomes with basic annotation. The process is presented for eleven specimens from two different insect subfamilies of medical importance to humans: Anophelinae (Diptera: Culicidae)—mosquitoes and Triatominae (Hemiptera: Reduviidae)—kissing bugs. Assembly of whole mitochondrial genome sequences of all 11 specimens along with the results of an ortholog search and BLAST against the NCBI nucleotide database are also presented.

## Introduction

Scientific collections document the diversity of life and preserve specimens to be studied by future generations. With modern advances in DNA sequencing techniques, museum specimens preserved for decades can continue to inform new investigations filling critical gaps in taxonomic knowledge. Since the advent of modern DNA sequencing techniques, there has been much debate over destructive sampling of specimens housed within scientific collections [1]. However, given the potential contributions that rare or understudied taxa can offer, the term “value-added” may be more appropriate [2]. Nonetheless, this distinction will probably depend on an individual curator’s viewpoint. Managers of scientific collections are more likely to put severe limits on destructive sampling of historical specimens [3], whereas evolutionary biologists may see scientific collections as a near endless resource of material ready to be tapped [4–6]. Thus, a balance must be struck between the value of genetic sampling while maintaining the integrity of these invaluable scientific collections.

Insect field collections represent considerable time and expense, and field biologists and taxonomists with the requisite expertise to build these collections are themselves becoming rare. Thus, access to rare or understudied taxa may only be possible through archive collections. With recent advancements in DNA sequencing, cryptic species—i.e., lineages that are morphologically indistinguishable but evolutionarily independent [7]—are playing a crucial role in our understanding of the evolutionary histories of medically important arthropod species. When assessment of reproductive isolation to determine species status is not feasible, researchers often defer to the phylogenetic species concept [8] to better understand the specific status of lineages of interest.

The development of extraction methods that solubilize and recover DNA from collection specimens—with little or no physical damage—was the first significant step in realizing the potential utility of historical samples in large-scale, molecular insect diversification studies [9,10]. However, only in the past decade random short read sequencing strategies (such as Illumina®) began to become more accessible. Before then, the most common and available method of DNA sequencing was Sanger sequencing [11], which relies on prior sequence knowledge, long DNA fragments, and the intact presence of the target gene region. These requirements of high-quality, un-sheared DNA for PCR amplification and Sanger sequencing served as a unsurmountable obstacle for air-dried, pinned insects, sometimes kept in environmentally unstable conditions [12], where the specimens’ DNA was likely to be highly degraded.

With the development of short read fragment sequencing platforms, such as Illumina®, obtaining partial genomes from degraded samples has become more straightforward, mainly because specific primer binding sites are not required, short fragmented DNA can be used for library construction, and *a priori* sequence knowledge is irrelevant. Here we document the combination of mostly non-destructive DNA extraction techniques and specific Illumina® library preparations that were fundamental in recovering both whole mitochondrial genomes and nuclear sequences from small and large collection specimens. Additionally, we describe the value of our e-vouchering pipeline to preserve and make available key taxonomic characters that may be destroyed through whole specimen sacrifice or otherwise damaged as a result of immersion in liquids (such as DNA extraction buffers). These methods enable the maximum value added from collection specimens.

The primary purpose of this work is to make available an accessible combination of methods that will allow any researchers, including those with limited expertise in genome sequencing and analysis, to be able to include archive specimens in their molecular systematics studies. The methodology described herein using archive specimens, can also be successfully applied to fresh specimens, and results in comparable data across all specimens.

## Materials and methods

### Sampling

Specimens examined during this study belong to two groups of medically important arthropod families: the Anophelinae (Diptera: Culicidae) [mosquitoes] and Triatominae (Hemiptera: Reduviidae) [kissing bugs]. These subfamilies are very different in size and physical reactions to the DNA extraction methodologies described below. Specimens were sourced from the U.S. National Culicidae and Heteroptera collections (Smithsonian Institution–National Museum of Natural History, Washington DC (USNM)).

Since mosquitoes are irreparably damaged by immersion in buffers (i.e., there is loss of scales and setae of vital taxonomic value), even mostly “non-destructive” DNA extraction protocols [3] are unusable for such samples. Thus, mosquito specimens chosen for DNA extraction and sequencing had to meet the four following criteria: (1) samples filled a critical taxonomic knowledge gap; (2) samples were in poor physical condition (i.e., of limited value to future taxonomic studies); (3) samples were part of a series of at least four specimens from the same collection event, with at least three other exemplars of the same sex; and (4) samples were from, or close to, the type locality.

Because the DNA extraction approach used here is non-destructive for Triatominae specimens, samples were randomly chosen from other ongoing studies.

A total of eleven specimens, five mosquitoes and six kissing bugs, collected between 1935 and 1998 were examined (Table 1).

**Table 1:** Specimens used in this study, their voucher number at USNM and the year of collection. Geographic origins are listed *verbatim* from the archive specimen labels.

Order Family Subfamily	Genus	Species	USNM Voucher	Year collected	Geographic Origin*
Diptera Culicidae Anophelinae	*Anopheles*	*albitarsis F*	USNMENT01222377	1998	Guárico, Venezuela
		*crucians B*	USNMENT01241735	1935	Brevard County, Florida, USA
		*ininii*	USNMENT01241795	1974	Pará, Brazil
		*maverlius*	USNMENT01241739	1975	Walton County, Florida, USA
	*Bironella*	*derooki*	USNMENT01241791	1944	Papua New Guinea
Hemiptera Reduviidae Triatominae	*Triatoma*	*dimidiata* s.l.	USNMENT01239007	1963	Yucatan, Mexico
		*dimidiata* s.l.	USNMENT01239008	1963	Costa Rica
		*dimidiata* s.l.	USNMENT01241973	1981	Borland’s cave, Toledo, Belize
		*dimidiata* s.l.	USNMENT01239010	1972	Colombia
		*dimidiata* s.l.	AMNH-IZC-00319805	1977	Panama
		*dimidiata* s.l.	USNMENT01241936	1981	Borland’s cave, Toledo, Belize

### E-vouchering

E-vouchers were generated for all mosquito samples destined for DNA extraction. These e-vouchers consist of a habitus image and images displaying the diagnostic characters for each species, as stated in the original description and subsequent taxonomic reviews. Original descriptions and taxonomic reviews were compiled for all Anophelinae specimens and reviewed for diagnostic morphological characters to capture during imaging [13–23]. Mosquito specimens were photographed prior to extraction at the NMNH Scanning Electron Microscopy Imaging Lab, using an Olympus DSX100 camera, and are available as electronic vouchers. Specimens were assigned a unique USNM catalog number along with a unique 2D barcode label. All images, sequence results and subsequent records are linked to the original museum specimen using this unique accession number. After DNA extraction, the Triatominae specimens were returned to the USNM collections and are available for further study. DNA extracts are vouchered in the NMNH biorepository.

### DNA extraction, sequencing and QC

DNA was extracted using the same protocol for both Anophelinae and Triatominae, since it is an established method for extraction of archive insect DNA, and eliminates the need for sample maceration. The method is largely non-destructive for larger specimens without scales, including Triatominae, and involves whole-specimen incubation in digestion buffer under gentle agitation for 16–20 hours. Culicidae were placed in 1.5ml tubes and Triatominae in 5 ml tubes with sufficient buffer for full immersion. After incubation, the ~4ml digestion solution for each Triatominae was divided into four separate 1.5ml tubes, which were processed independently for the DNA precipitation, using Sodium chloride and Glycoblue coprecipitant (ThermoFisher Scientific, Waltham, MA, 15 mg/mL diluted to 10ul/ml of digestion buffer) as previously described [3]. The detailed protocol, based on Gilbert et al. [4] is available at https://wrbu.si.edu/docs/sops/MolLabSOP1.pdf.

DNA was quantified for all samples using the High Sensitivity kit for Qubit Fluorometric Quantification (ThermoFisher Scientific, Waltham, MA). In cases where the DNA concentration was too low to quantify, the DNA solution was concentrated by evaporation using a Savant SpeedVac Plus Centrifuge Vacuum Concentrator and re-quantified.

Illumina© library prep was performed using KAPA HyperPlus Kits, (Roche, Pleasanton, CA). Since archive specimens exhibit highly fragmented DNA as verified by Agilent TapeStation 4200 Automated Electrophoresis (Agilent Technologies, Blacksburg, VA), the library prep began at the DNA end repair and A-tailing steps following the manufacturer’s protocol. Adapter ligation and PCR amplification conditions followed the manufacturer’s recommendations based on the quantity of DNA available. Subsequent quality control and fragment distribution were again assessed with the TapeStation 4200 (Agilent Technologies) and AMPure XP beads (Beckman Coulter, Sykesville, MD) clean-up was performed to remove adapter dimers and other impurities, when necessary. The detailed protocol, based on the KAPA HyperPlus technical datasheet is available at https://wrbu.si.edu/docs/sops/MolLabSOP2.pdf.

Sequencing was performed either using the HiSeq® Illumina® platform (PE 2x150) at Omega Bioservices (Norcross, GA, USA) [Triatominae], or NovaSeq® Illumina® platform (PE 2x150) [Culicidae] at the Walter Reed Army Institute of Research (WRAIR). Sequencing at Omega Bioservices was done as fee for service, for which 10 gigabases (Gb) of raw reads per sample were purchased. For the NovaSeq platform, the samples were run on a S4 flow cell with XP workflow as part of a bigger project, designed to maximize the overall raw reads output. Read quality was assessed using fastqc [24] and adapter trimming and sequence filtering was performed using Trimmomatic [7].

### Reference guided assembly

The mitochondrial genome was recovered using reference guided assembly. Reference sequences used for the mitochondrial genomes were, respectively: *Triatoma dimidiata* (GenBank NC_002609) and *Anopheles cruzii* (GenBank NC_024740). However, this is a highly customizable step in the pipeline described herein, and any available genomic region can be used as reference.

Trimmed and filtered paired reads, were mapped to their respective reference genome using bowtie2 [25], and writing only the mapped reads to the sequence alignment map (SAM) file output. Consensus sequences of the mapped reads were generated using Unipro UGENE v. 35 [26].

### De novo assembly and annotation of nuclear genome and ortholog search

Paired sequence files output by Trimmomatic were used as input for the GATB-Minia Pipeline (https://github.com/GATB/minia) for whole genome assembly. Assembly statistics were calculated using abyss-fac [27] and annotation was performed using AUGUSTUS [28], with the provided training sets for Triatomine and Anophelinae, *Rhodnius* and *Aedes*, respectively. Outputs were processed using getAnnoFasta.pl (github.com/nextgenusfs/augustus/blob/master/scripts/getAnnoFasta.pl).

Orthograph [29] databases were constructed using the OrthoDB v. 10.1 [30] set of single-copy orthologs (SCO) for Hemiptera and Diptera, following a custom pipeline (available at https://github.com/jsoghigian/orthoset_construction). Coding sequences output from AUGUSTUS were used as input for the search of SCO. All identified SCO were blasted against the NCBI database, using BLASTn (nucleotide query, nucleotide database) and BLASTp (amino acid query, amino acid database) [31] and the first hit was recorded. Hits were then compared to NCBI taxonomy database, and the number of arthropod orthologs was recorded for each specimen.

In order to identify variable correlations with the quality of the assembly/recovered orthologs, Pearson correlation amongst result variables was calculated using the function ggscatter of the ggpubr R [32] package. The code used is available as supporting information (S1 Appendix).

All the scripts and commands used perform the de novo assembly, annotation and ortholog calling, BLASTn and BLASTp results filtering are publicly available (https://github.com/silviajusti/publications/blob/master/SCO_pipeline.md). The pipeline includes all the analytical steps described on the methods. This includes selection and output of identified by BLAST as belonging to the target group (in this case Arthropods).

## Results

### DNA extraction and sequencing

Total DNA extracted ranged from 21 to 7379 ng per specimen, which generated 0.6–10 gigabases (Gb) of raw Illumina sequencing results for all five archive mosquitoes and six kissing bug specimens (Table 2). Although complete mitochondrial genomes were uniformly assembled, nuclear genomes varied from two genomes with over 2,000 SCO identified by Orthograph, to specimens with only a few SCO. Details are below. Obtained raw reads are available on GenBank as SRA project PRJNA646392.

**Table 2:** Amount of total genomic DNA extracted and amount of raw data (i.e., raw reads in gigabases– 10^9^ bp) obtained per specimen.

Specimen	Total DNA extracted (ng)	Raw sequence data obtained (Gb)
*An*. *albitarsis* F USNMENT01222377	11	2
*An*. *crucians* B USNMENT01241735	20	3
*An*. *Ininii* USNMENT01241795	5	10
*An*. *maverlius* USNMENT01241739	95	0.6
*Bi*. *derooki* USNMENT01241791	6	3
*T*. *dimidiata* s.l. USNMENT01239007	2774	10
*T*. *dimidiata* s.l. USNMENT01239008	7379	10
*T*. *dimidiata* s.l. USNMENT01241973	2222	10
*T*. *dimidiata* s.l. USNMENT01239010	2126	10
*T*. *dimidiata* s.l. AMNH-IZC-00319805	2232	10
*T*. *dimidiata* s.l. USNMENT01241936	2232	10

### Reference guided assembly

Regardless of the collection date of the specimen (Triatomines: 1963–1981; Culicids: 1935–1998) or the amount of total DNA recovered, it was possible to assemble and annotate the complete mitochondrial genomes for all 11 archive samples (Table 3). The size of the mitochondrial genomes assembled for the Anophelinae specimens varied between 15,390 bp and 15,430 bp, while the Triatominae mitochondrial genomes assembled ranged between 16,398 and 17,283 bp. All known mitochondrial genes were present in all recovered mitochondrial genomes, and the gene organization recovered was the same as the references.

**Table 3:** Recovered mitochondrial genome sizes and their respective GenBank accession numbers.

Specimen	Mitochondrial genome size	GenBank accession (Mitochondrial Genome)
*An*. *albitarsis* F USNMENT01222377	15,424 bp	MT757854
*An*. *crucians* B USNMENT01241735	15,404 bp	MT757853
*An*. *ininii* USNMENT01241795	15,390 bp	MT757855
*An*. *maverlius* USNMENT01241739	15,430 bp	MT426121
*Bi*. *derooki* USNMENT01241791	15,407 bp	MT757856
*T*. *dimidiata* s.l. USNMENT01239007	17,283 bp	MT733873
*T*. *dimidiata* s.l. USNMENT01239008	17,276 bp	MT757848
*T*. *dimidiata* s.l. USNMENT01241973	16,398 bp	MT757849
*T*. *dimidiata* s.l. USNMENT01239010	17,267 bp	MT757850
*T*. *dimidiata* s.l. AMNH-IZC-00319805	17,019 bp	MT757851
*T*. *dimidiata* s.l. USNMENT01241936	16,571 bp	MT733872

### De novo assembly

De novo assembly of the sequences for each specimen was performed without previous filtering for contaminants, in order to be able to allow Orthograph [29] to assemble the genomes based on the constructed reference database alone. Direct assemblies of trimmed and filtered reads were assessed for contiguity using abyss-fac [27]. Both the highest and the lowest N50 (i.e., shortest contig of 50% of the assembly length) were observed on the Triatominae samples: 631-bp (*T*. *dimidiata* USNMENT01241973) and 2619-bp (*T*. *dimidiata* USNMENT01239008). Anophelinae samples also showed extremes: N50 ranged between 640-bp (*An*. *albitarsis* F USNMENT01222377) and 2189-bp (*An*. *maverlius* USNMENT01241739).

Unlike the N50 values, the highest and the lowest sum of the sequence sizes were found for the Anophelinae samples. Interestingly, the lowest sum of contig lengths was observed for *An*. *maverlius*, the sample with the highest N50 (see Table 4 for more details on the assembly statistics).

**Table 4:** Summary statics for the genome assembly of each specimen: Total contigs in the assembly (N), contiguity of the assembly (N50), sum of contig lengths (SUM).

ID	N	N50	SUM
*An*. *albitarsis* F USNMENT01222377	1.13E+05	6.40E+02	1.00E+07
*An*. *crucians* B USNMENT01241735	1.83E+03	8.52E+02	1.41E+05
*An*. *ininii* USNMENT01241795	1.87E+05	1.72E+03	1.49E+08
*An*. *maverlius* USNMENT01241739	1.88E+02	2.19E+03	3.12E+04
*Bi*. *derooki* USNMENT01241791	4.70E+04	1.98E+03	1.84E+07
*T*. *dimidiata* s.l. USNMENT01239007	3.09E+04	1.15E+03	2.01E+06
*T*. *dimidiata* s.l. USNMENT01239008	1.48E+04	2.62E+03	1.98E+06
*T*. *dimidiata* s.l. USNMENT01241973	1.53E+05	6.31E+02	1.23E+07
*T*. *dimidiata* s.l. USNMENT01239010	2.56E+03	1.15E+03	2.81E+05
*T*. *dimidiata* s.l. AMNH-IZC-00319805	2.40E+03	1.28E+03	1.89E+05
*T*. *dimidiata* s.l. USNMENT01241936	1.78E+04	1.45E+03	1.47E+06

### Ortholog search, BLAST and QC

The constructed Orthograph databases comprise 1,709 and 3,612 SCO for Hemiptera and Diptera, herein used as reference for Triatominae and Anophelinae specimens, respectively. Out of these, only five to 253 SCO were recovered for the six Triatominae specimens, compared to one to 3,470 SCO for the five Culicidae specimens (Table 5). BLAST results for the recovered SCO showed that not all orthologs identified by Orthograph correspond to known Arthropod sequences. BLASTp results always returned a higher number of hits than BLASTn, likely due to the size difference between nr and nt databases.

**Table 5:** Comparison between the number of SCO identified by Orthograph, the total number of BLASTn hits, the total number of BLASTn hits that correspond to known Arthropod sequences, the total number of BLASTp hits, the total number of BLASTp hits that correspond to known Arthropod sequences.

Specimen	Number of SCO recovered	Total number of BLASTn hits	Number of BLASTn Arthropods hits	Total number of BLASTp hits	Number of BLASTp Arthropods hits
*An*. *albitarsis* F USNMENT01222377	2209	947	776	2201	2188
*An*. *crucians* B USNMENT01241735	14	10	4	14	6
*An*. *ininii* USNMENT01241795	3470	2229	1973	3367	3367
*An*. *maverlius* USNMENT01241739	1	1	1	1	1
*Bi*. *derooki* USNMENT01241791	235	165	50	233	24
*T*. *dimidiata* s.l. USNMENT01239007	59	22	9	58	40
*T*. *dimidiata* s.l. USNMENT01239008	83	35	11	82	16
*T*. *dimidiata* s.l. USNMENT01241973	253	64	28	252	228
*T*. *dimidiata* s.l. USNMENT01239010	5	4	1	5	1
*T*. *dimidiata* s.l. AMNH-IZC-00319805	19	18	8	19	2
*T*. *dimidiata* s.l. USNMENT01241936	40	30	9	40	12

Pearson correlations were reciprocally calculated for the following variables: specimen age (calculated based on collection date), total DNA extracted (ng), number of SCO identified by Orthograph, total contigs in the assembly (N), contiguity of the assembly (N50), sum of contig lengths (SUM), number of arthropod hits with BLASTn, number of arthropod hits with BLASTp.

Variables that were found to be positively and significantly correlated (p<0.05) were the number of SCO identified by Orthograph, N, N50, SUM, number of arthropod hits with BLASTn, number of arthropod hits with BLASTp (Table 6). No variables were found to be negatively correlated and, the age of the specimen was not significantly correlated to any of the other variables.

**Table 6:** Pearson correlations calculated between the variables.

	Age	Total DNA (ng)	SCO	N	N50	SUM	BLASTn
Total DNA (ng)	0.054						
SCO	-0.29	-0.38					
N	-0.27	-0.27	0.8*				
N50	0.1	0.39	-0.096	-0.28			
SUM	-0.034	-0.3	0.85*	0.73*	0.13		
BLASTn	-0.21	-0.35	0.98*	0.76*	-0.0042	0.94*	
BLASTp	-0.32	-0.37	1*	0.79*	-0.12	0.84*	0.97*

Age: Based on collection date; Total DNA (ng): Total amount of genomic DNA extracted from the specimens; SCO: Number of single-copy orthologs identified by Orthograph; N: Total contigs in the assembly; N50: Shortest contig length contained on the first half of the assembly (i.e., with the largest group of contigs); SUM: Sum of contig lengths; BLASTn: Number of arthropod hits with BLASTn; BLASTp: Number of arthropod hits with BLASTp. * Indicates significant correlations with p<0.05.

When the recovered orthologs were blasted against the NCBI nucleotide (nt) and protein (nr) databases, with only the first hit being recorded, there was an almost perfect positive correlation between the number of orthologs recovered and the number of arthropod hits (Table 6). Lower quality assemblies (i.e., low number SCO recovered), usually yielded a higher percentage of contaminant microorganism blast hits [data available at doi.org/10.25573/data.12659711].

## Discussion

In this study we describe a successful combination of established wet laboratory protocols, with a bioinformatics pipeline that allowed for the recovery of complete mitochondrial genome sequences from all insect specimens, which had been previously collected 20–84 years ago (the latter, *An*. *crucians* B). This is remarkable considering the raw sequence data differed by over an order of magnitude and there was a large range in the number of nuclear SCO recovered (with only one SCO for one specimen). Despite being developed specifically for archive specimens, the Walter Reed Biosystematics Unit standard operating procedure (SOP) described here can also be applied to freshly collected, frozen, dried or ethanol-stored specimens.

The results presented highlight the advantage of the non-targeted short read DNA sequencing by reflecting the quality and availability of the starting material, avoiding long and laborious experimental hours in the search for genomic regions that might just be too degraded to be recovered by Sanger Sequencing [22] or by targeted enrichment approaches [21]. Specimen age (calculated based on collection date) also appears irrelevant to the results obtained, increasing the utility of archive insect collections for genetic studies. Personal field-to-lab observation by the authors lead to the hypothesis that the preserving procedure to which each specimen is subjected prior to being vouchered and deposited in the collection, rather than age, are likely very important factors.

It is incredible to observe that, regardless of specimen age or insect size, this methodology allowed for the assembly of complete mitochondrial genomes for all the dry, pinned specimens studied, compounded with the advantage of extracting nuclear SCO, and possibly other nuclear genes of interest, depending on the reference sequence used.

Illustrating the lack of correlation between the age of the specimen and the quality of the sequencing results, the highest number of SCO was recovered from a specimen 42 years old (*An*. *ininii–* 3,367 arthropod SCO), a better result than for the most recent specimen (*An*. *albitarsis* F– 2,188 arthropod SCO). Specimen size, and consequently amount of DNA extracted, was not a predictor of assembly contiguity or recovery of orthologs. For the much larger triatomines, only 0.05–13.3% of all arthropod SCO from the Hemiptera database were recovered; while for the mosquitoes between 0.02–93.2% of all arthropod SCO from the Diptera database were recovered. In addition, multicopy genes (e.g., nuclear ribosomal genes), which were not identified here, may be useful for phylogenetic analysis. While the mitochondrial genome was used here as an example for the reference guided assembly, in future applications, any genomic region can be used as reference. It is important to note that, in this case, results will depend on sequenced genomic DNA availability.

The BLAST results, however, highlight the need for caution with the Orthograph [29] recovered SCO. While Orthograph has been widely used to identify putative orthologous groups [33–36], it also can infer putative homology even from distantly related species. As such, contaminants that are not cleaned from assemblies may be misidentified as orthologs in the organism of interest (e.g., see blast results for the *T*. *dimidiata* s.l. specimens). Particularly in the case of museum specimens, where contaminant DNA (e.g., fungi) could be as abundant as target DNA, care should be taken to verify the identity of contigs in assemblies or Orthograph putative orthologs, against a larger database of nucleotide or protein sequences, such as with NCBI BLAST.

The use of insect archive specimens for phylogenetic studies is by no means a new endeavor. In fact, researchers have been including such specimens in systematics or evolutionary studies for almost two decades now [5,37–43]. While preserving the integrity of the specimen is essential, in most cases a whole leg was used for DNA extraction, which may or may not have impacted downstream taxonomic character availability, but also may not have provided sufficient starting quantities of DNA for downstream analyses. Use of a specimen portion highlights the importance of e-vouchering, even when the specimen is kept virtually intact.

While the expectation of a contiguous, close to complete genome as the standard of genomic projects, low-coverage genomes are sufficient for both deep and shallow phylogenomic studies [44–46]. Moreover, it is worth noting that even partial genomes may offer data for a wide range of future projects regardless of method of capture, unlike sequence capture (AHE/UCE) [38] where the data captured is limited to the probes, which can be extremely limited in their taxonomic coverage/utility.

Finally, financial savings on labor might just be greater than experiment-related savings provided by the use of these less costly approaches, with the added bonus of additional data. Even though the study of archive specimens is often associated with systematics and evolutionary biology, the usefulness of such specimens, especially for disease vectors and agricultural pests, can go beyond basic science and help understand other important factors, such as the evolution of insecticide resistance [39].

Scientific collections offer a unique resource for investigating the evolutionary history of extant and extinct taxa. Rare or cryptic taxa, not typically available among freshly collected specimens, can fill critical gaps in knowledge. However, there is a trade-off to having scientific collections available to inform future investigations. The approach outlined herein attempts to balance the need to apply modern DNA sequencing tools to understudied/rare taxa and the need to preserve voucher specimens. Whenever possible, non-destructive methods should be used for sampling scientific collections. When destructive sampling is unavoidable, samples should be part of a related series and careful attention should be paid to document any sacrificed specimen. E-vouchers provide the opportunity for morphology to be examined retrospectively in the context of sequencing and phylogenetic analysis results, even when the physical specimen is lost or damaged in the process.

## Conclusion

Regardless of the fact that this study is based upon DNA extracted from Arthropods, the pipeline described is applicable to any starting DNA, provided that taxa-specific steps (e.g., DNA extraction methodology, orthograph database and BLAST filters) are adjusted accordingly.

Additionally, the variation of the results observed, even for the specimens with comparable sequencing depths, leads to the conclusion that the starting DNA (i.e., physical availability of the genome regions in the sample) is the single most important factor for the recovery of genome coverage. The quality of the starting DNA will be a direct result of handling and storage of the specimen, from the field to its final destiny.

## Supporting information

S1 Appendix R code and results for the pairwise correlation comparison of the variables studied. (PDF)
