# Supplementary material for: From e-voucher to genomic data: Preserving archive specimens as demonstrated with medically important mosquitoes (Diptera: Culicidae) and kissing bugs (Hemiptera: Reduviidae)
Source: PLoS One. 2021 Feb 25;16(2):e0247068. doi: 10.1371/journal.pone.0247068 (PMC7906454; doi:10.1371/journal.pone.0247068)
Supplement: S1 Appendix — (PDF) [file pone.0247068.s001.pdf]

Testing Correlations

Silvia Justi

4/30/2020

```
ggscatter(my_data, x = "age", y = "Total.DNA.extracted..ng.",
          add = "reg.line", conf.int = TRUE,
          cor.coef = TRUE, cor.method = "pearson")
```

```
## `geom_smooth()` using formula 'y ~ x'

## Warning: Removed 11 rows containing non-finite values (stat_smooth).

## Warning: Removed 11 rows containing non-finite values (stat_cor).

## Warning: Removed 11 rows containing missing values (geom_point).
```

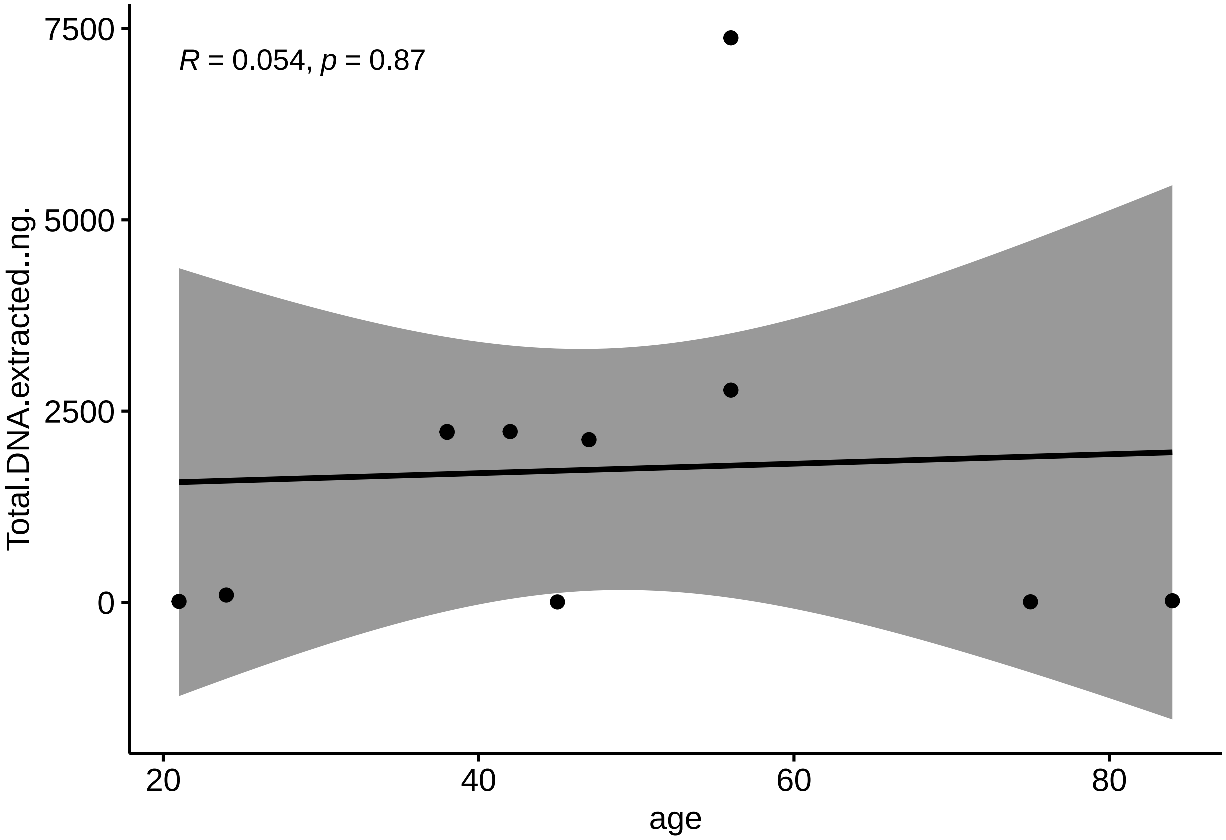

```r
ggscatter(my_data, x = "age", y = "SCO_number",
          add = "reg.line", conf.int = TRUE,
          cor.coef = TRUE, cor.method = "pearson")
```

```
## `geom_smooth()` using formula 'y ~ x'

## Warning: Removed 11 rows containing non-finite values (stat_smooth).

## Warning: Removed 11 rows containing non-finite values (stat_cor).

## Warning: Removed 11 rows containing missing values (geom_point).
```

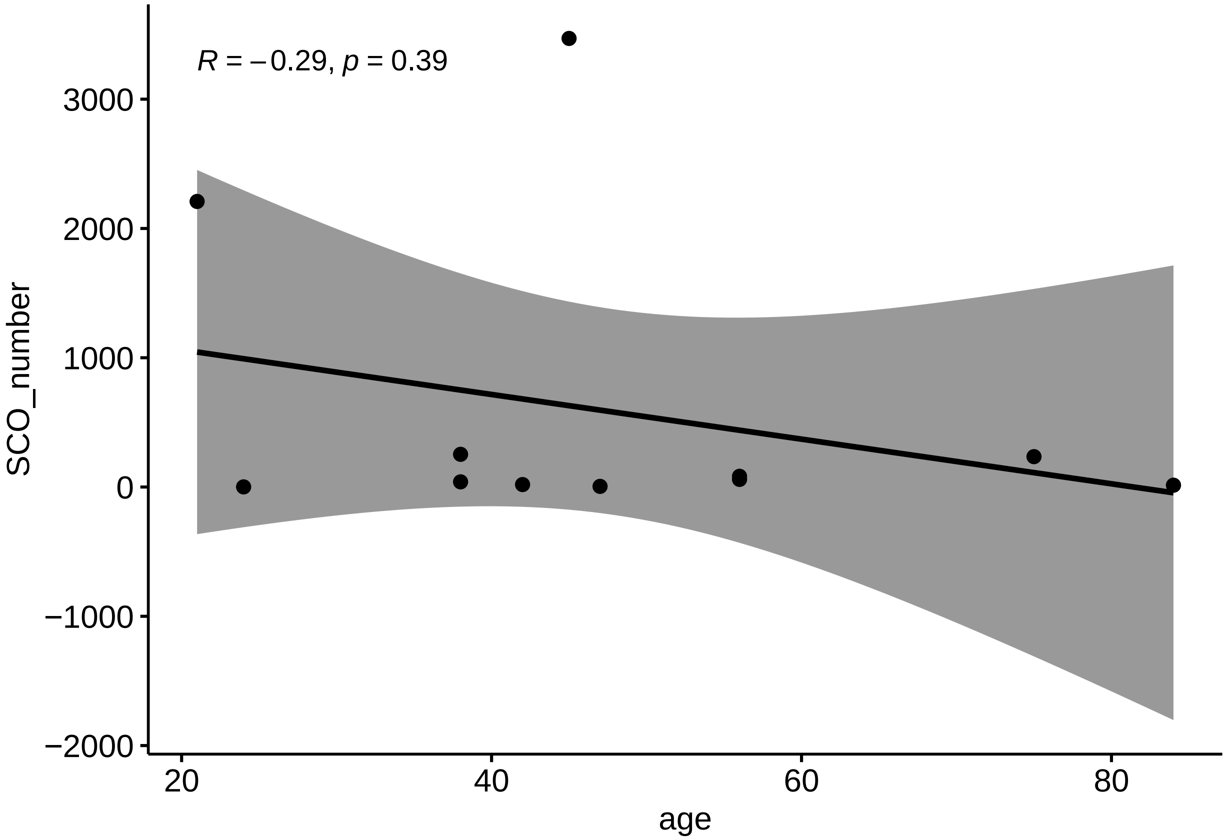


```r
ggscatter(my_data, x = "age", y = "n",
          add = "reg.line", conf.int = TRUE,
          cor.coef = TRUE, cor.method = "pearson")
```

```
## `geom_smooth()` using formula 'y ~ x'

## Warning: Removed 11 rows containing non-finite values (stat_smooth).

## Warning: Removed 11 rows containing non-finite values (stat_cor).

## Warning: Removed 11 rows containing missing values (geom_point).
```

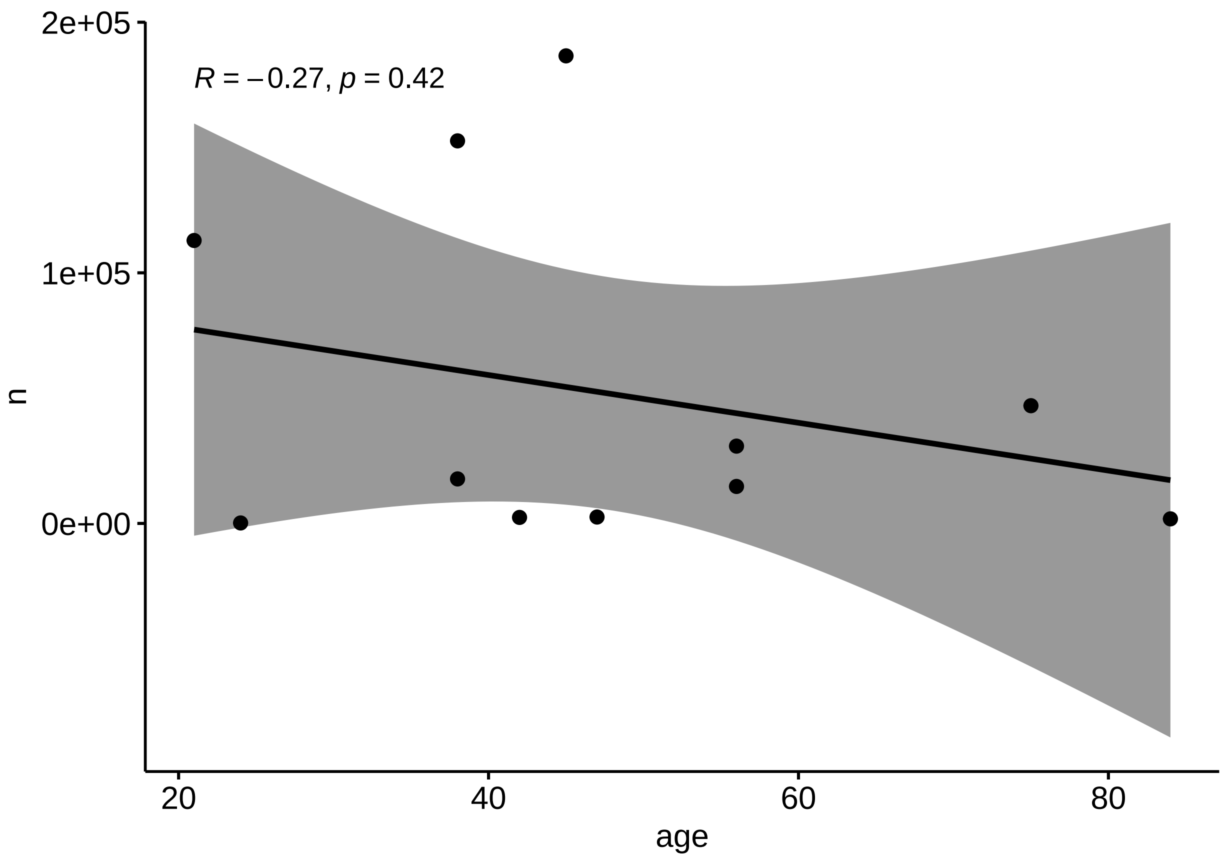


```r
ggscatter(my_data, x = "age", y = "N50",
          add = "reg.line", conf.int = TRUE,
          cor.coef = TRUE, cor.method = "pearson")
```

```
## `geom_smooth()` using formula 'y ~ x'

## Warning: Removed 11 rows containing non-finite values (stat_smooth).

## Warning: Removed 11 rows containing non-finite values (stat_cor).

## Warning: Removed 11 rows containing missing values (geom_point).
```

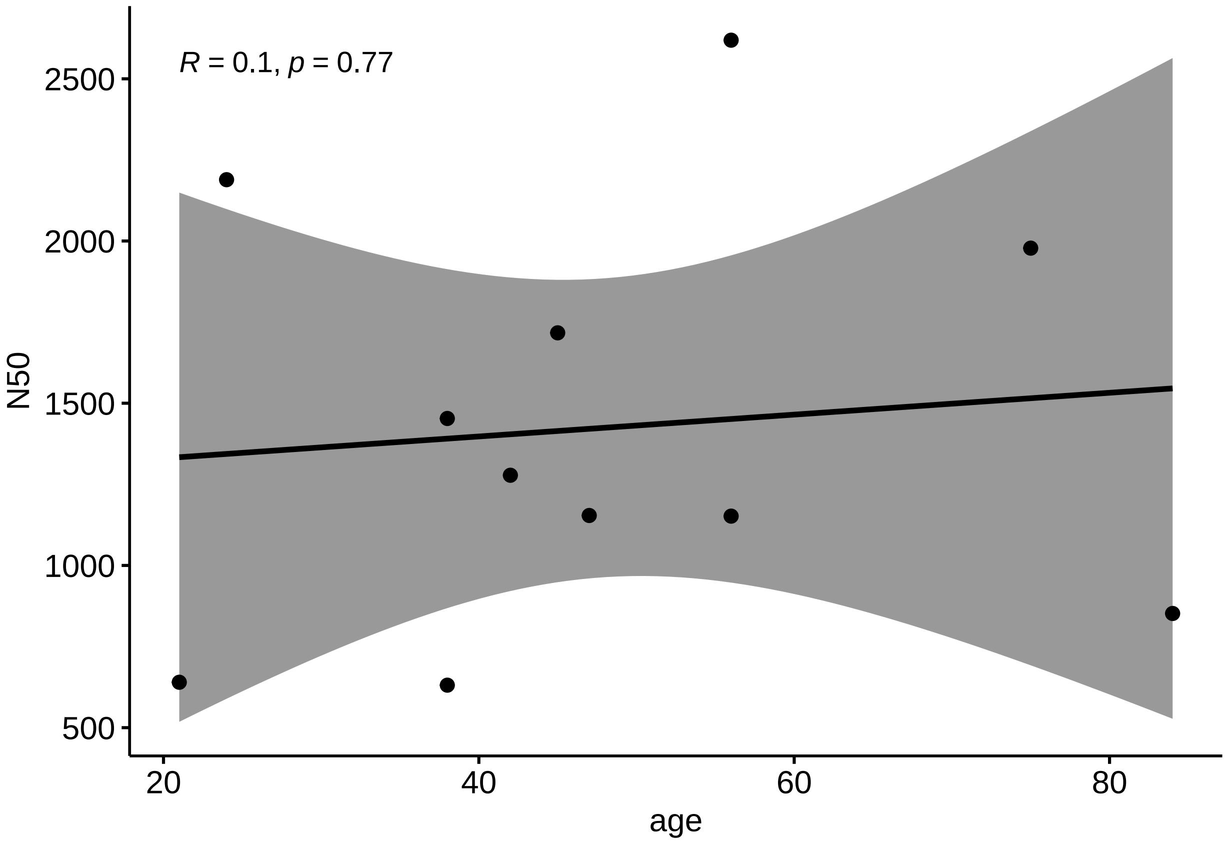


```
ggscatter(my_data, x = "age", y = "sum",
          add = "reg.line", conf.int = TRUE,
          cor.coef = TRUE, cor.method = "pearson")
```

```
## `geom_smooth()` using formula 'y ~ x'

## Warning: Removed 11 rows containing non-finite values (stat_smooth).

## Warning: Removed 11 rows containing non-finite values (stat_cor).

## Warning: Removed 11 rows containing missing values (geom_point).
```

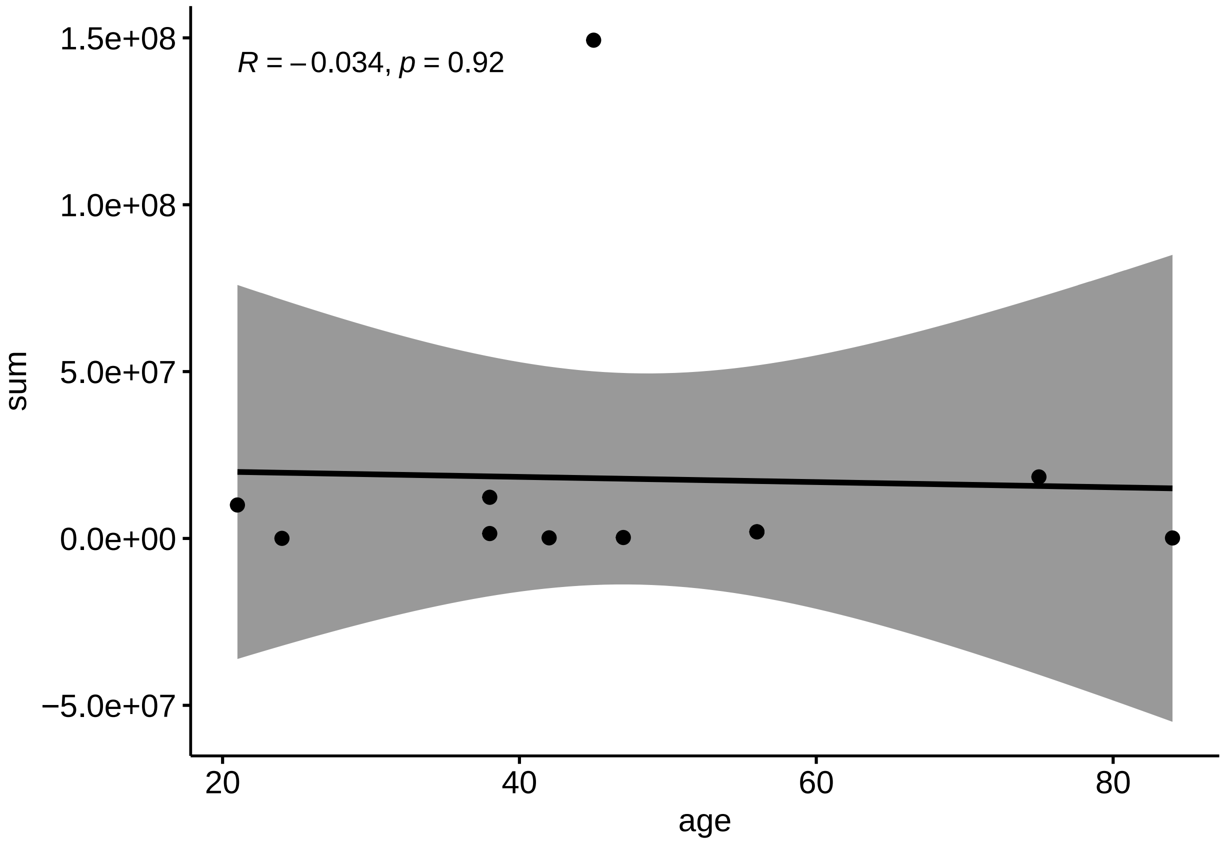


```
ggscatter(my_data, x = "age", y = "BlastnSCO",
          add = "reg.line", conf.int = TRUE,
          cor.coef = TRUE, cor.method = "pearson")
```

```
## `geom_smooth()` using formula 'y ~ x'

## Warning: Removed 11 rows containing non-finite values (stat_smooth).

## Warning: Removed 11 rows containing non-finite values (stat_cor).

## Warning: Removed 11 rows containing missing values (geom_point).
```

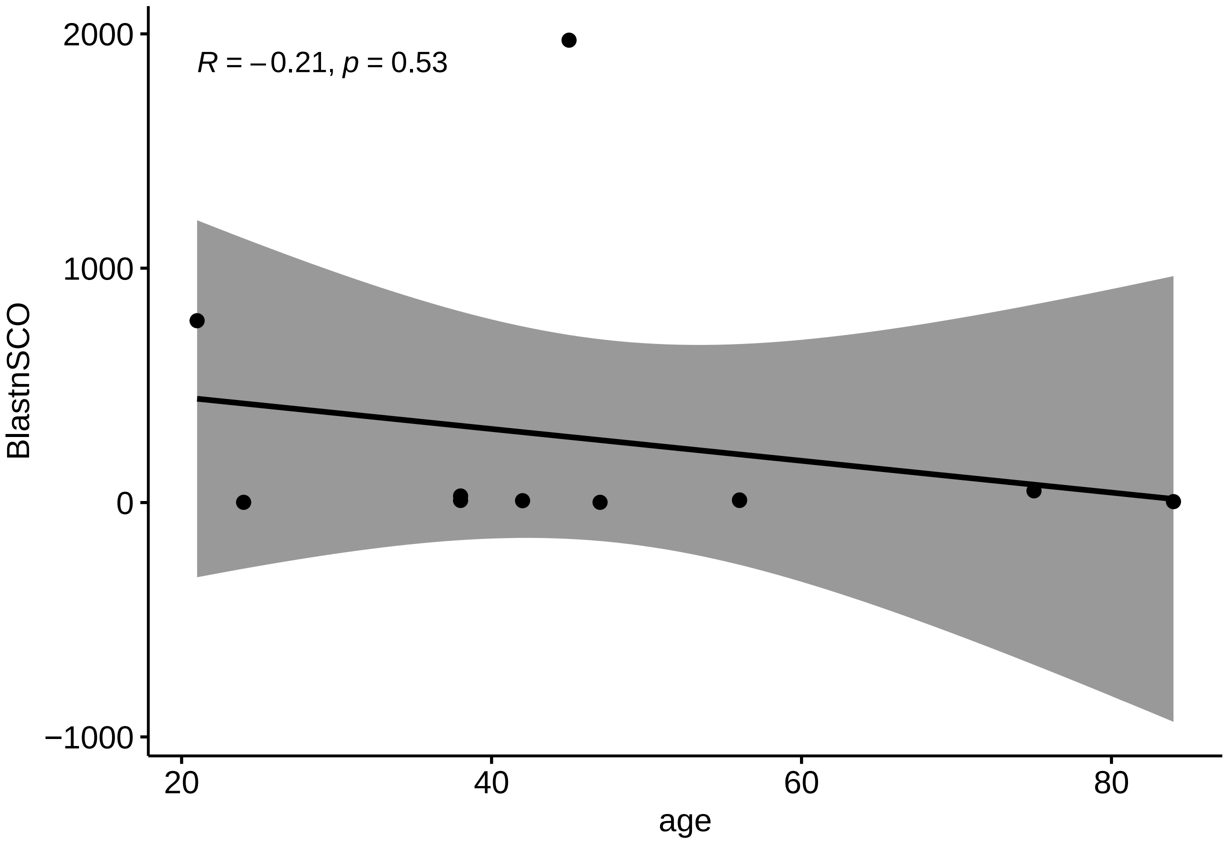


```r
ggscatter(my_data, x = "age", y = "BlastpSCO",
          add = "reg.line", conf.int = TRUE,
          cor.coef = TRUE, cor.method = "pearson")
```

```
## `geom_smooth()` using formula 'y ~ x'

## Warning: Removed 11 rows containing non-finite values (stat_smooth).

## Warning: Removed 11 rows containing non-finite values (stat_cor).

## Warning: Removed 11 rows containing missing values (geom_point).
```

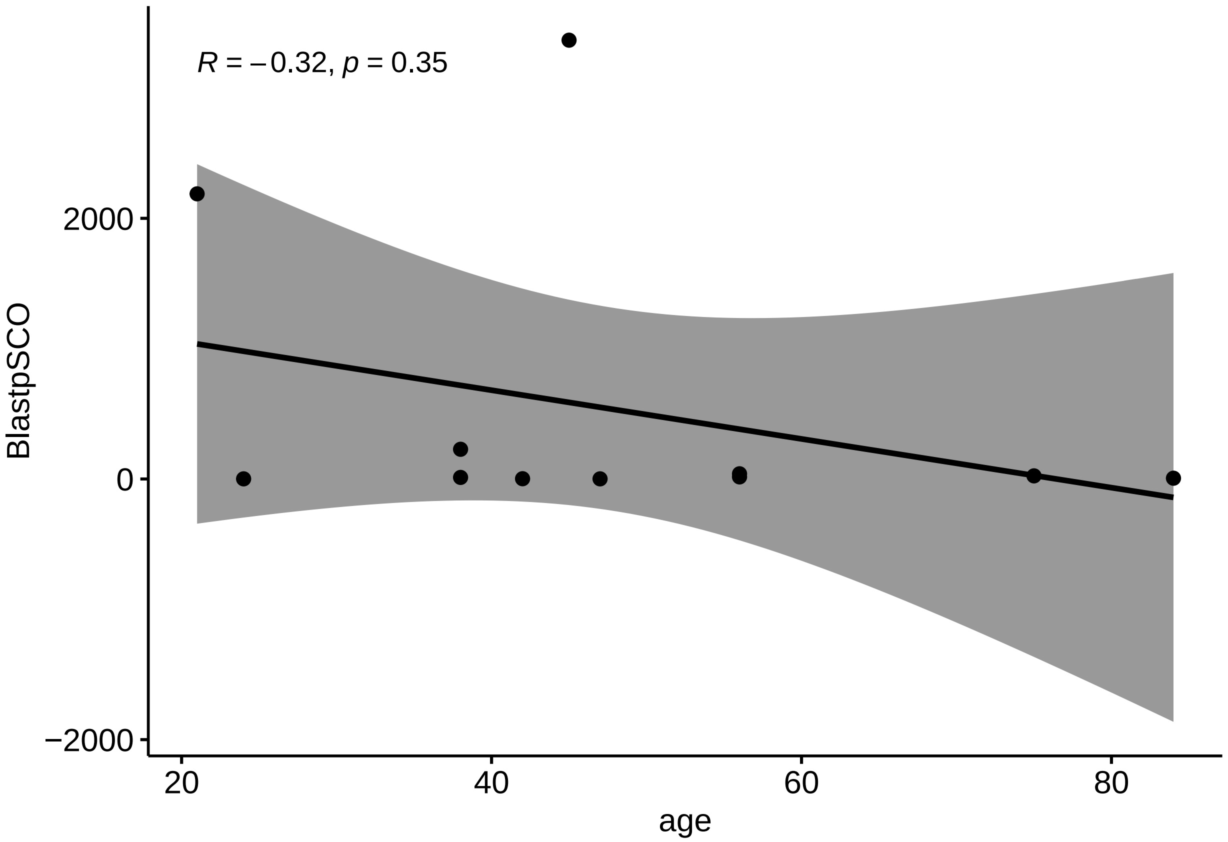


```r
ggscatter(my_data, x = "Total.DNA.extracted..ng.", y = "SCO_number",
          add = "reg.line", conf.int = TRUE,
          cor.coef = TRUE, cor.method = "pearson")
```

```
## `geom_smooth()` using formula 'y ~ x'

## Warning: Removed 11 rows containing non-finite values (stat_smooth).

## Warning: Removed 11 rows containing non-finite values (stat_cor).

## Warning: Removed 11 rows containing missing values (geom_point).
```

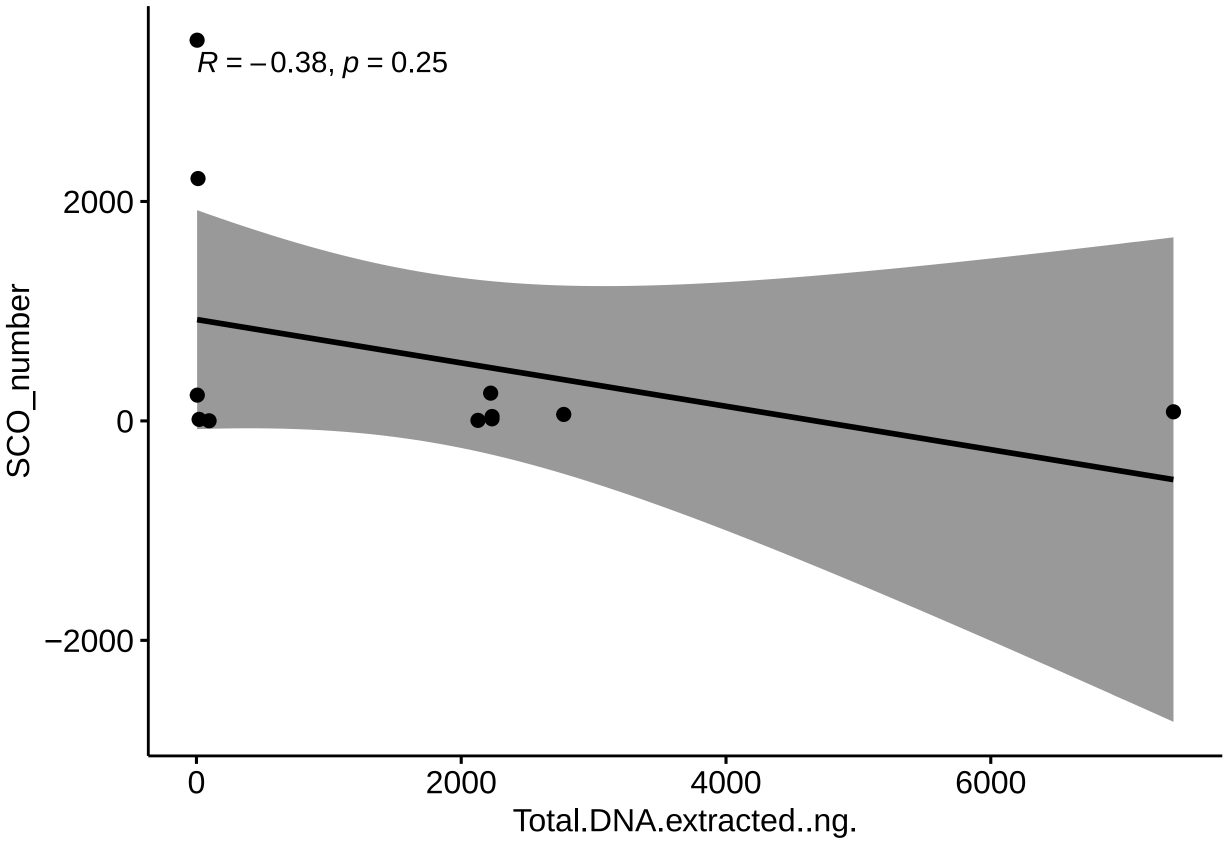


```
ggscatter(my_data, x = "Total.DNA.extracted..ng.", y = "n",
          add = "reg.line", conf.int = TRUE,
          cor.coef = TRUE, cor.method = "pearson")
```

```
## `geom_smooth()` using formula 'y ~ x'

## Warning: Removed 11 rows containing non-finite values (stat_smooth).

## Warning: Removed 11 rows containing non-finite values (stat_cor).

## Warning: Removed 11 rows containing missing values (geom_point).
```

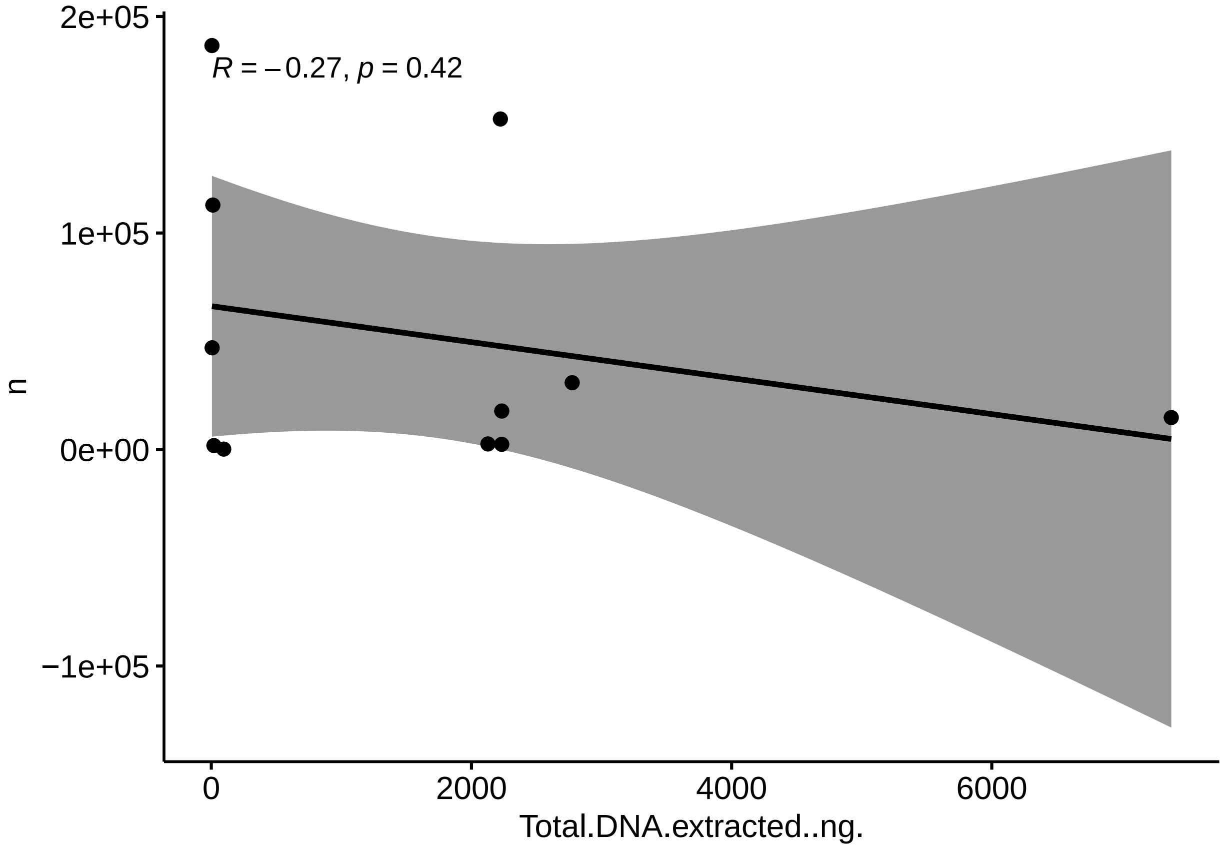


```
ggscatter(my_data, x = "Total.DNA.extracted..ng.", y = "N50",
          add = "reg.line", conf.int = TRUE,
          cor.coef = TRUE, cor.method = "pearson")
```

```
## `geom_smooth()` using formula 'y ~ x'

## Warning: Removed 11 rows containing non-finite values (stat_smooth).

## Warning: Removed 11 rows containing non-finite values (stat_cor).

## Warning: Removed 11 rows containing missing values (geom_point).
```

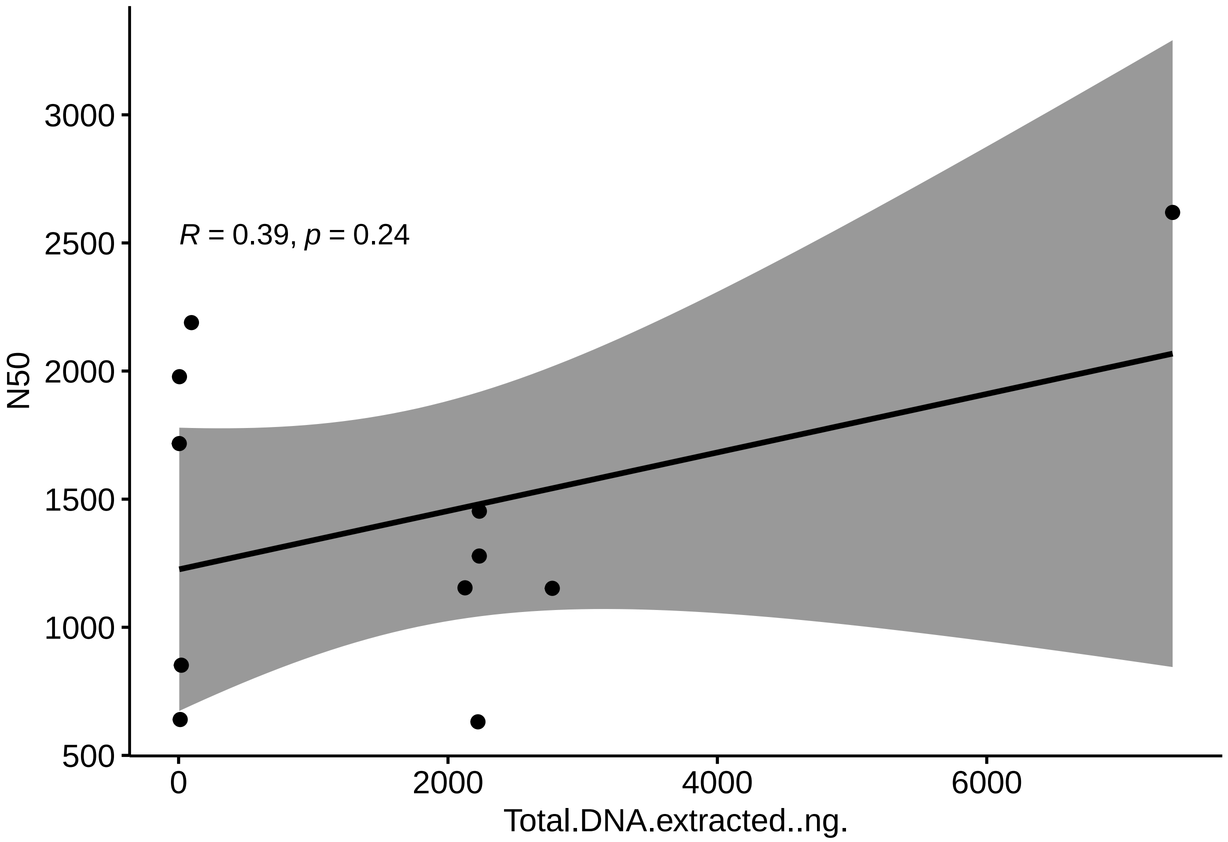


```r
ggscatter(my_data, x = "Total.DNA.extracted..ng.", y = "sum",
          add = "reg.line", conf.int = TRUE,
          cor.coef = TRUE, cor.method = "pearson")
```

```
## `geom_smooth()` using formula 'y ~ x'
```

```
## Warning: Removed 11 rows containing non-finite values (stat_smooth).
```

```
## Warning: Removed 11 rows containing non-finite values (stat_cor).
```

```
## Warning: Removed 11 rows containing missing values (geom_point).
```

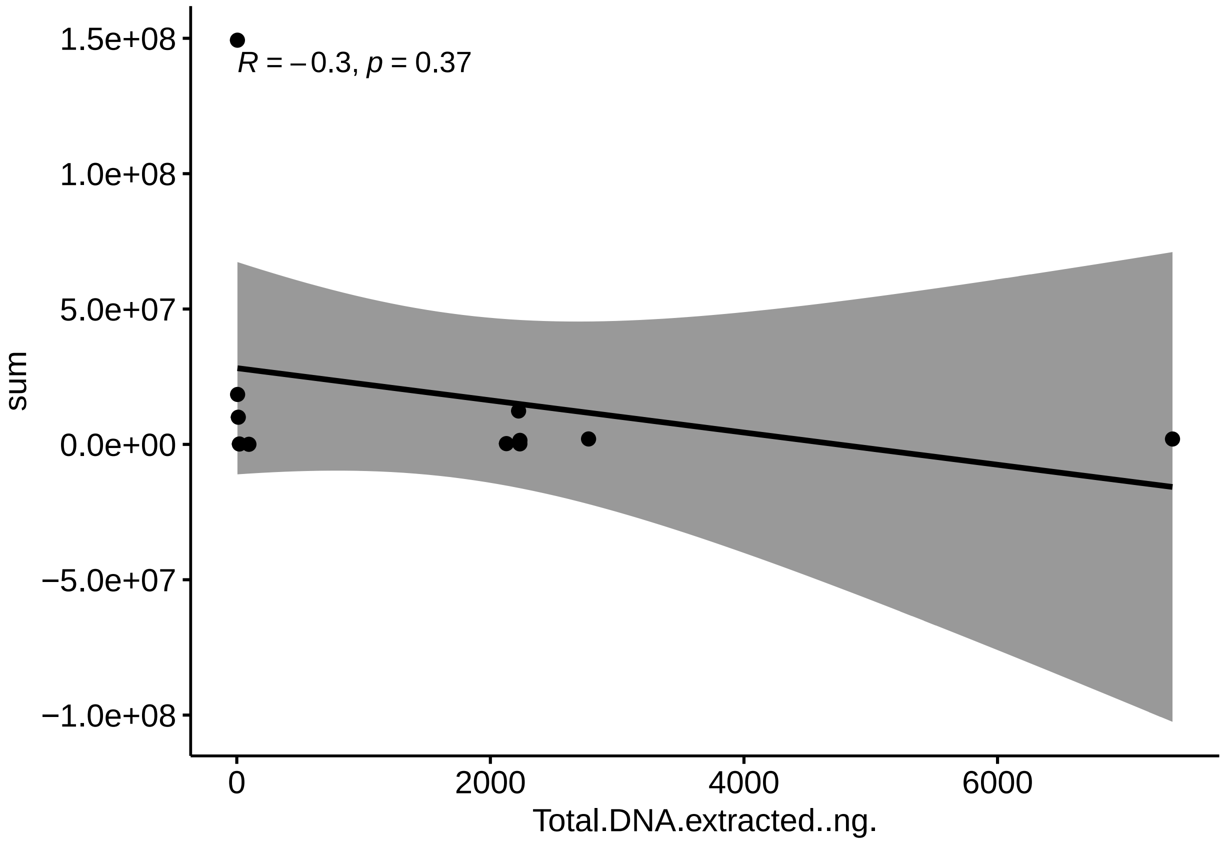


```
ggscatter(my_data, x = "Total.DNA.extracted..ng.", y = "BlastnSCO",
          add = "reg.line", conf.int = TRUE,
          cor.coef = TRUE, cor.method = "pearson")
```

```
## `geom_smooth()` using formula 'y ~ x'

## Warning: Removed 11 rows containing non-finite values (stat_smooth).

## Warning: Removed 11 rows containing non-finite values (stat_cor).

## Warning: Removed 11 rows containing missing values (geom_point).
```

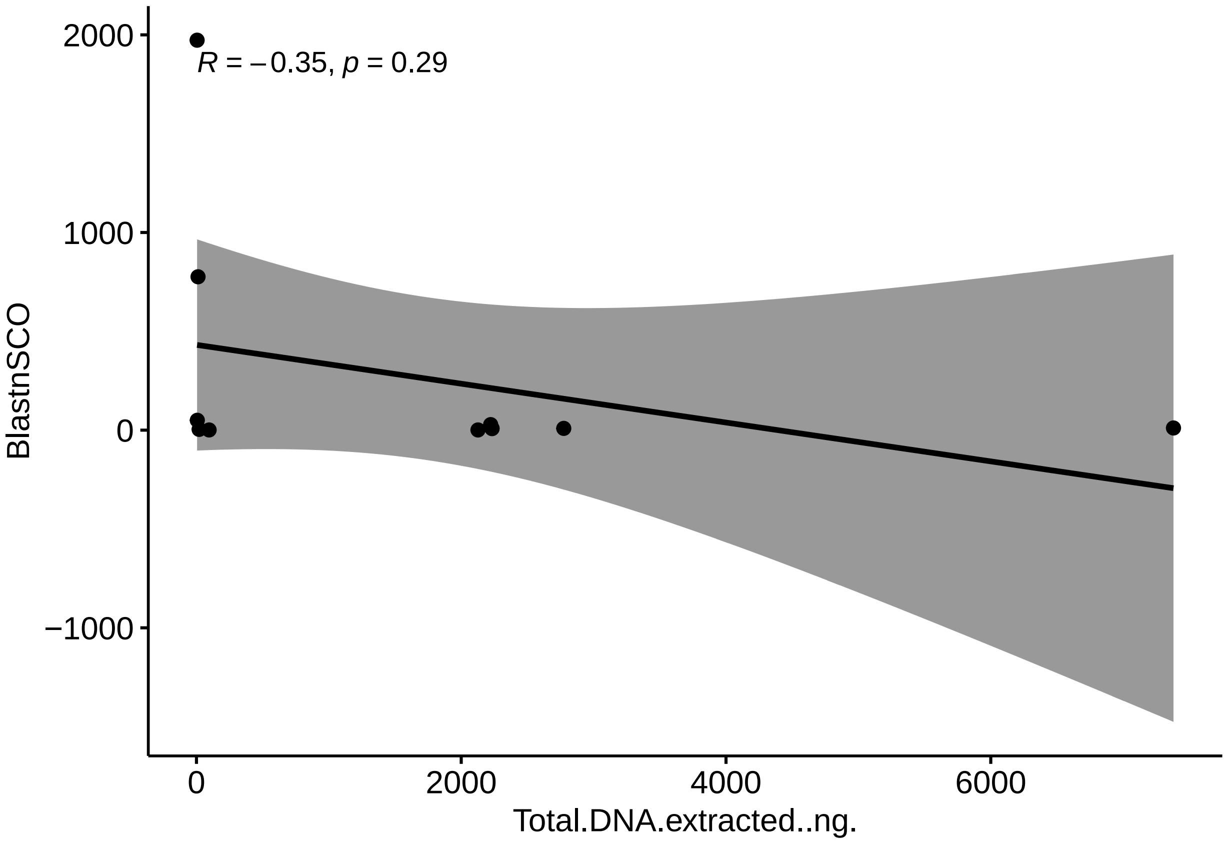


```r
ggscatter(my_data, x = "Total.DNA.extracted..ng.", y = "BlastpSCO",
          add = "reg.line", conf.int = TRUE,
          cor.coef = TRUE, cor.method = "pearson")
```

```
## `geom_smooth()` using formula 'y ~ x'
```

```
## Warning: Removed 11 rows containing non-finite values (stat_smooth).
```

```
## Warning: Removed 11 rows containing non-finite values (stat_cor).
```

```
## Warning: Removed 11 rows containing missing values (geom_point).
```

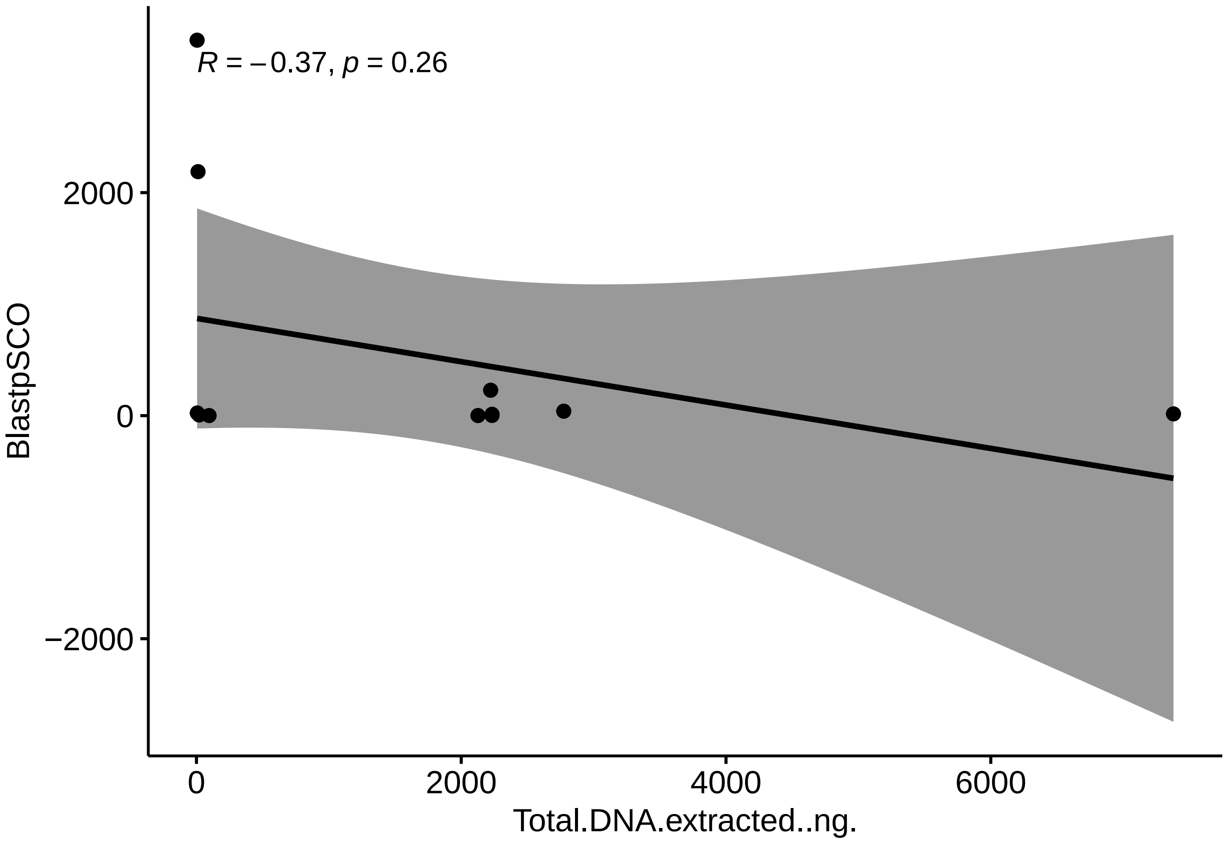


```r
ggscatter(my_data, x = "SCO_number", y = "n",
          add = "reg.line", conf.int = TRUE,
          cor.coef = TRUE, cor.method = "pearson")
```

```
## `geom_smooth()` using formula 'y ~ x'

## Warning: Removed 11 rows containing non-finite values (stat_smooth).

## Warning: Removed 11 rows containing non-finite values (stat_cor).

## Warning: Removed 11 rows containing missing values (geom_point).
```

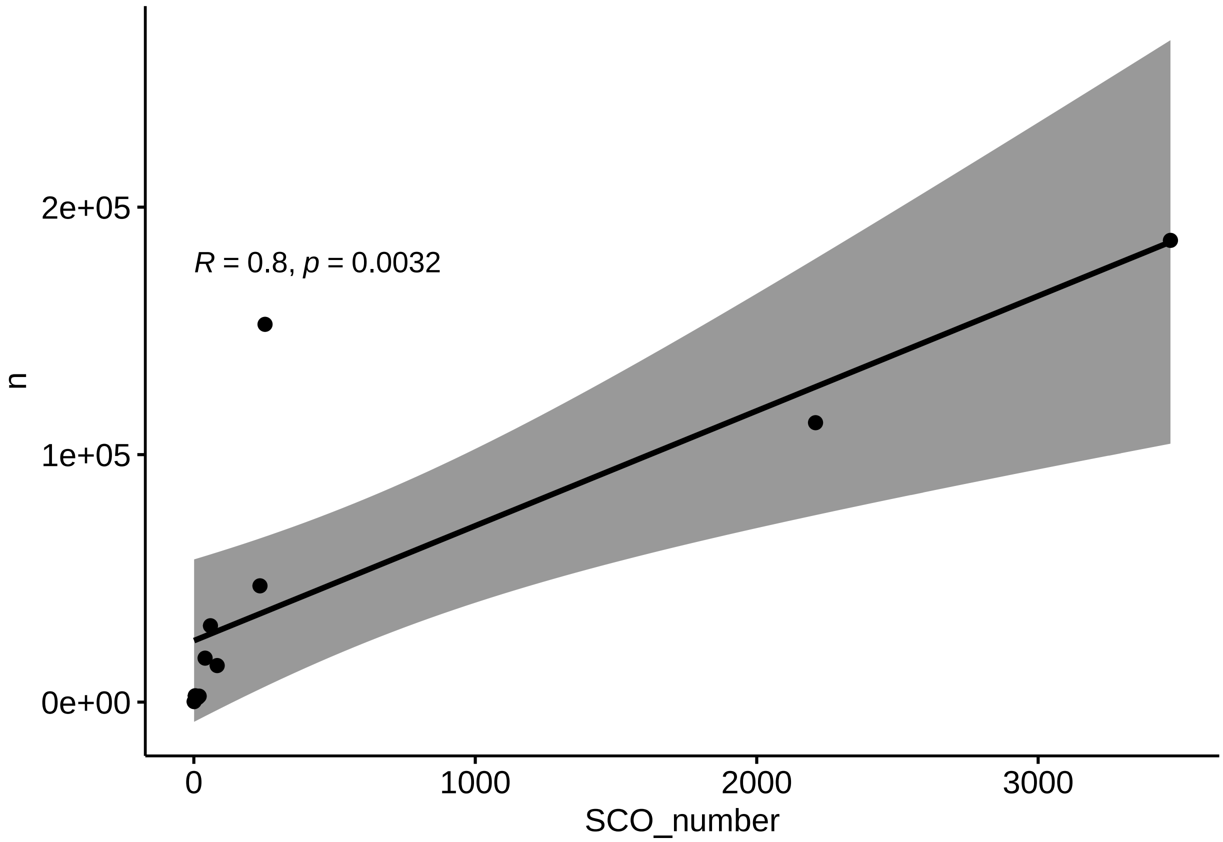


```
ggscatter(my_data, x = "SCO_number", y = "N50",
          add = "reg.line", conf.int = TRUE,
          cor.coef = TRUE, cor.method = "pearson")
```

```
## `geom_smooth()` using formula 'y ~ x'

## Warning: Removed 11 rows containing non-finite values (stat_smooth).

## Warning: Removed 11 rows containing non-finite values (stat_cor).

## Warning: Removed 11 rows containing missing values (geom_point).
```

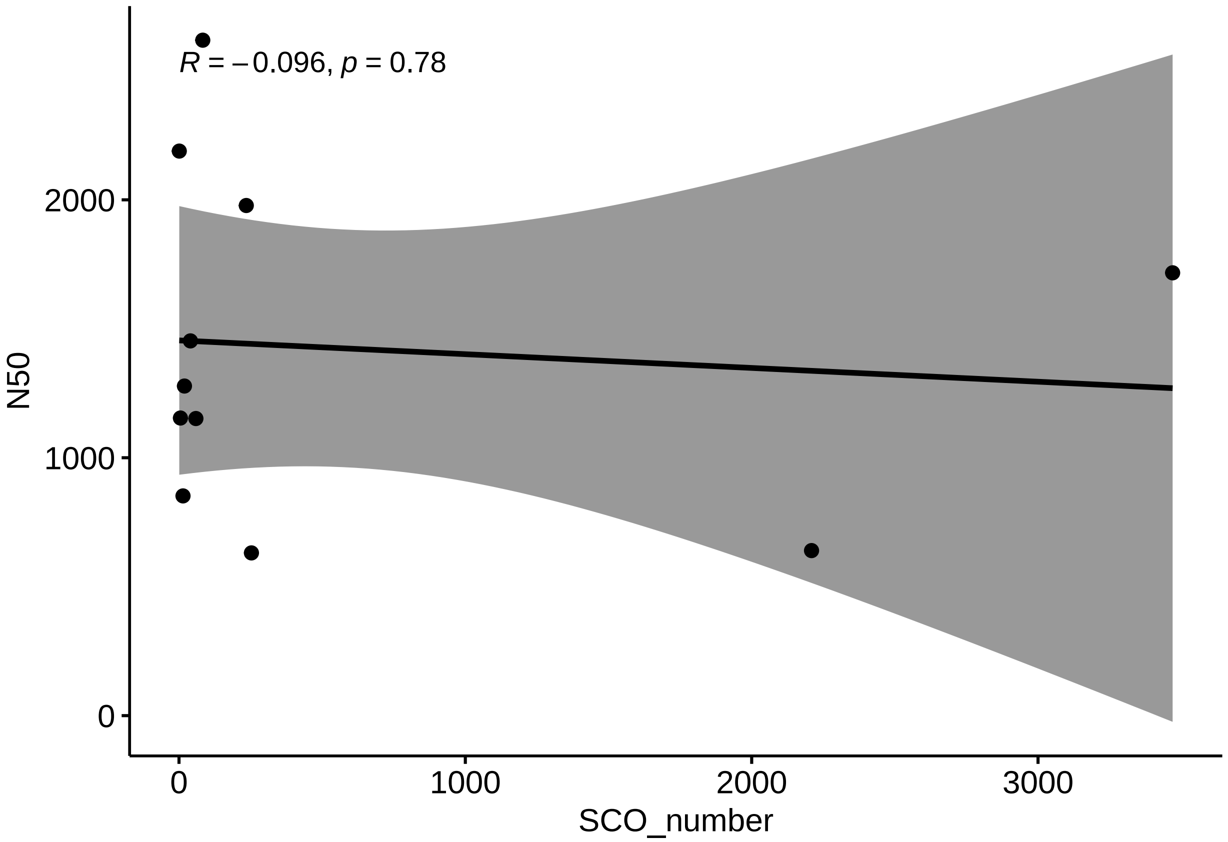


```
ggscatter(my_data, x = "SCO_number", y = "sum",
          add = "reg.line", conf.int = TRUE,
          cor.coef = TRUE, cor.method = "pearson")
```

```
## `geom_smooth()` using formula 'y ~ x'

## Warning: Removed 11 rows containing non-finite values (stat_smooth).

## Warning: Removed 11 rows containing non-finite values (stat_cor).

## Warning: Removed 11 rows containing missing values (geom_point).
```

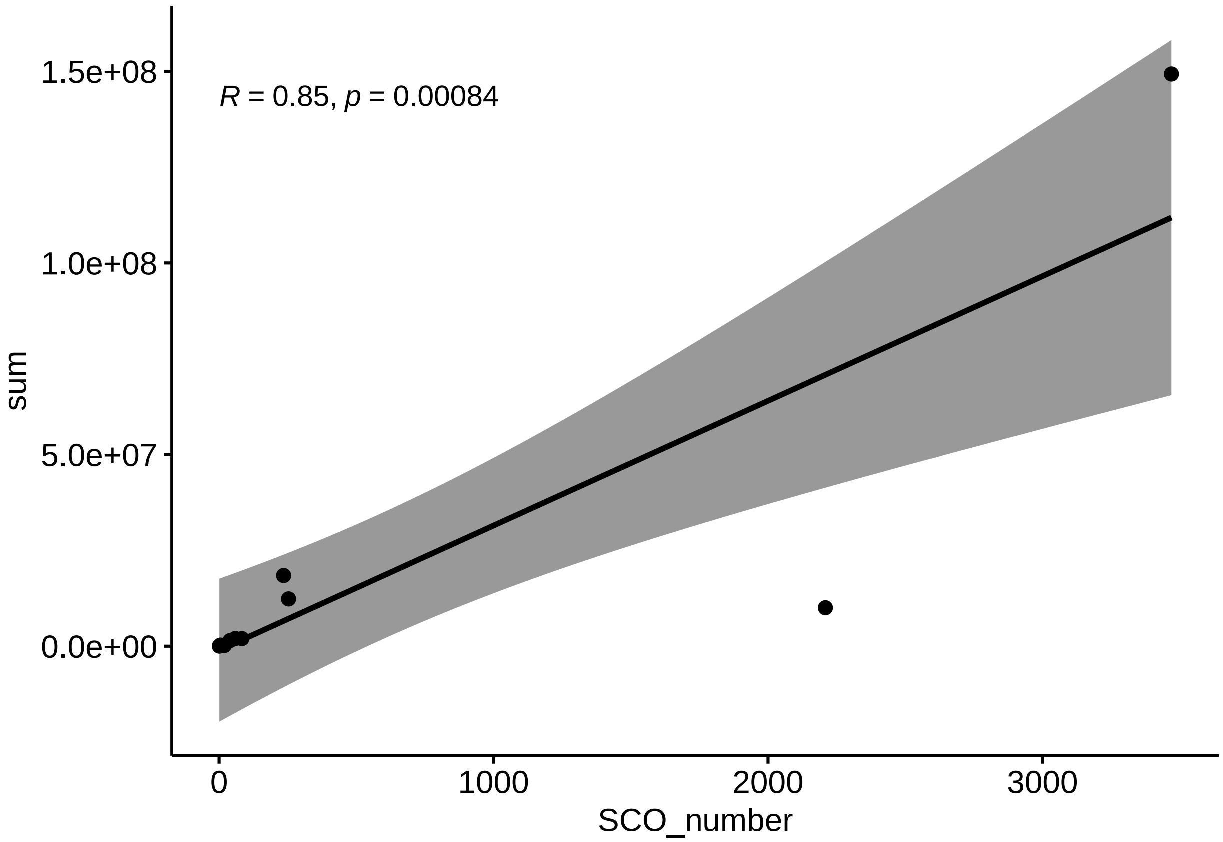


```
ggscatter(my_data, x = "SCO_number", y = "BlastnSCO",
          add = "reg.line", conf.int = TRUE,
          cor.coef = TRUE, cor.method = "pearson")
```

```
## `geom_smooth()` using formula 'y ~ x'
```

```
## Warning: Removed 11 rows containing non-finite values (stat_smooth).
```

```
## Warning: Removed 11 rows containing non-finite values (stat_cor).
```

```
## Warning: Removed 11 rows containing missing values (geom_point).
```

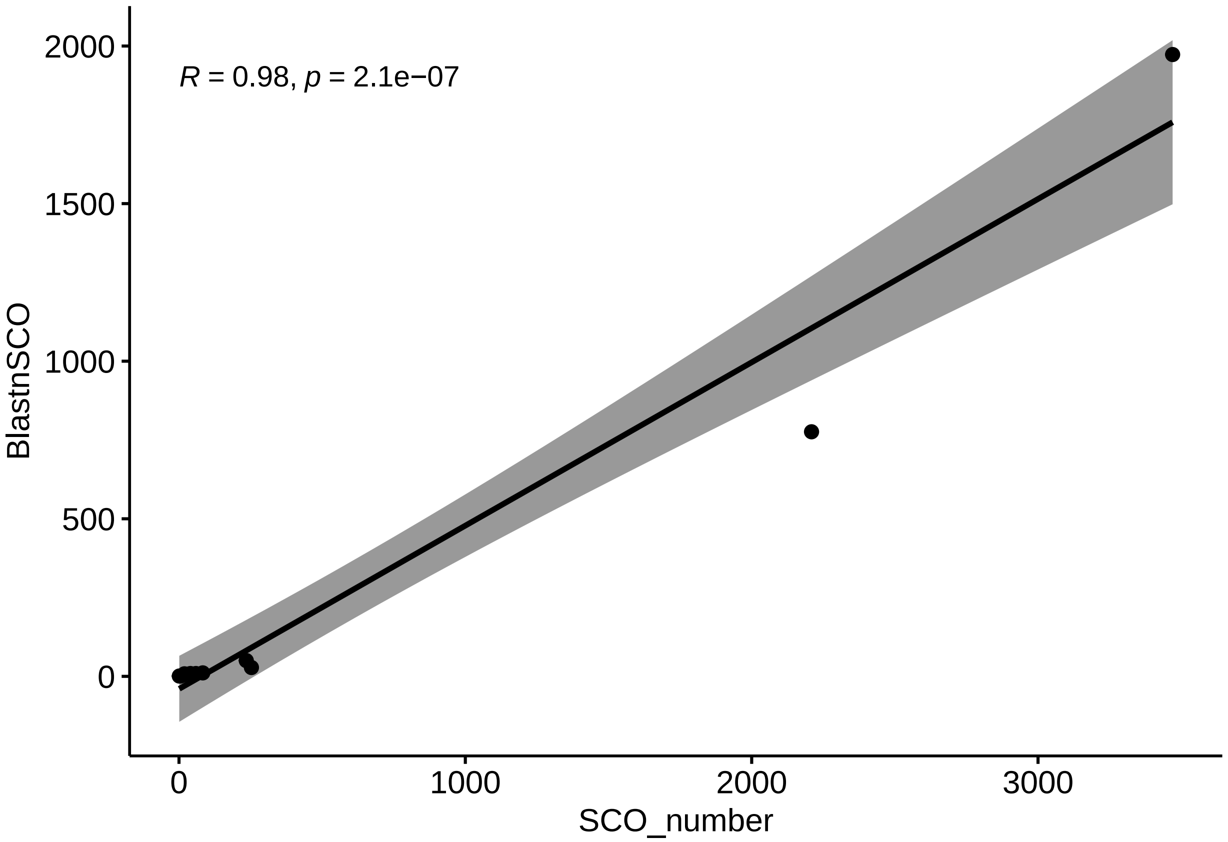


```r
ggscatter(my_data, x = "SCO_number", y = "BlastpSCO",
          add = "reg.line", conf.int = TRUE,
          cor.coef = TRUE, cor.method = "pearson",
          xlab = "SCO_number", ylab = "# Blastp Arthropod")
```

```
## `geom_smooth()` using formula 'y ~ x'

## Warning: Removed 11 rows containing non-finite values (stat_smooth).

## Warning: Removed 11 rows containing non-finite values (stat_cor).

## Warning: Removed 11 rows containing missing values (geom_point).
```

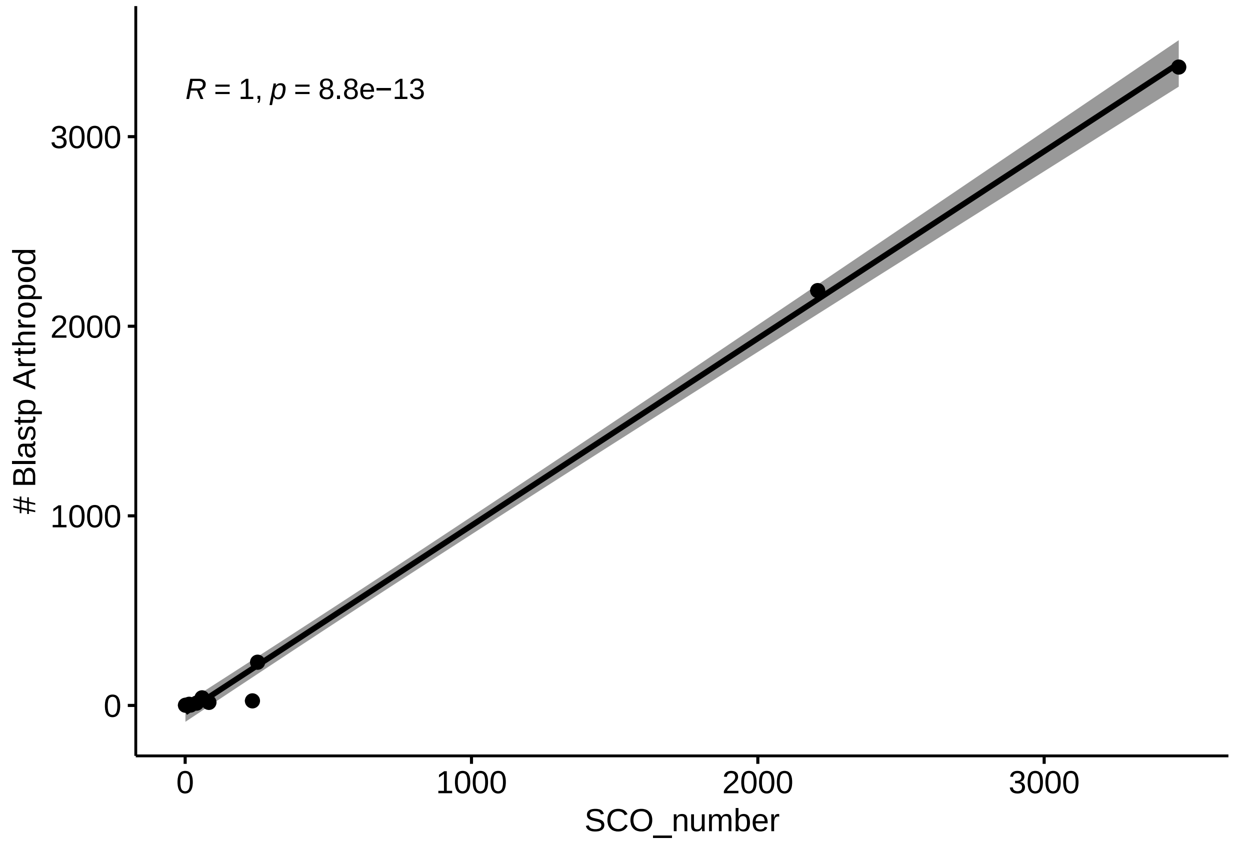


```r
ggscatter(my_data, x = "n", y = "N50",
          add = "reg.line", conf.int = TRUE,
          cor.coef = TRUE, cor.method = "pearson")
```

```
## `geom_smooth()` using formula 'y ~ x'
```

```
## Warning: Removed 11 rows containing non-finite values (stat_smooth).
```

```
## Warning: Removed 11 rows containing non-finite values (stat_cor).
```

```
## Warning: Removed 11 rows containing missing values (geom_point).
```

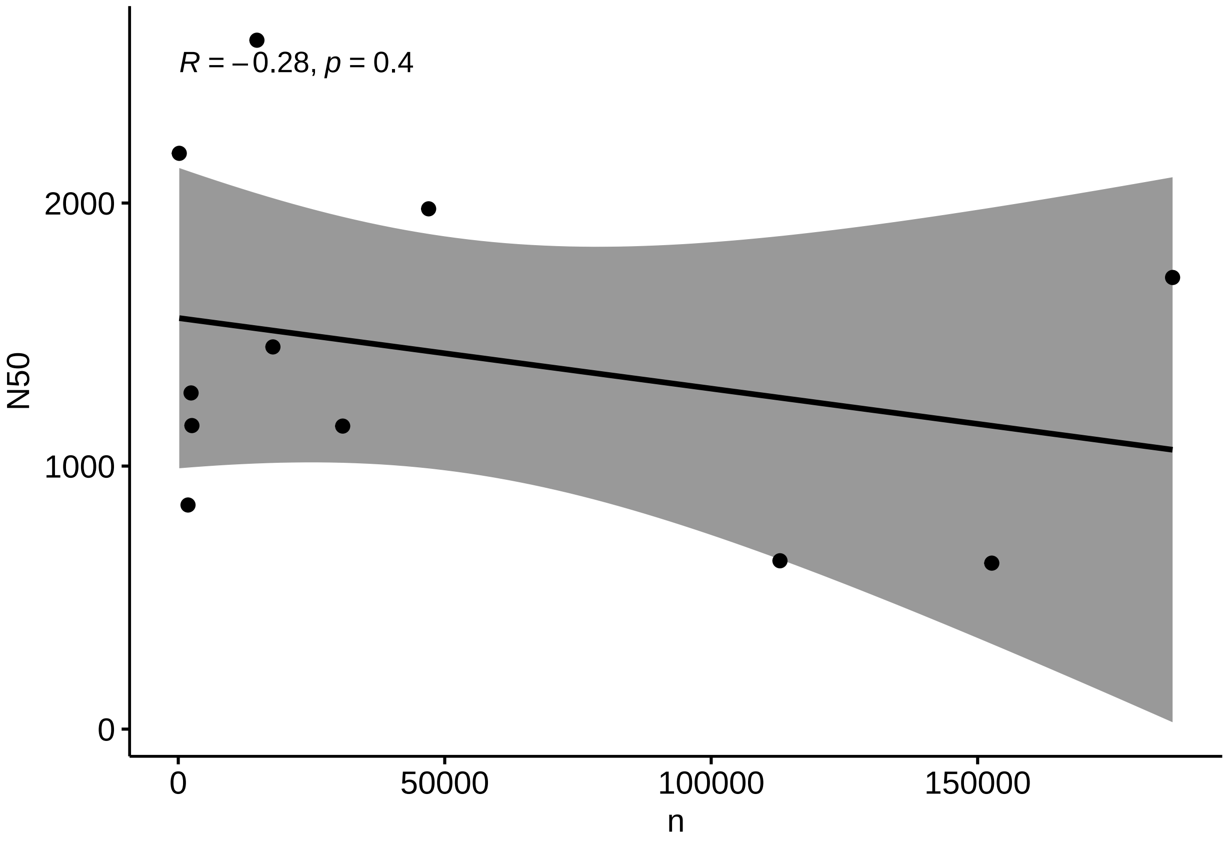


```r
ggscatter(my_data, x = "n", y = "sum",
          add = "reg.line", conf.int = TRUE,
          cor.coef = TRUE, cor.method = "pearson")
```

```
## `geom_smooth()` using formula 'y ~ x'

## Warning: Removed 11 rows containing non-finite values (stat_smooth).

## Warning: Removed 11 rows containing non-finite values (stat_cor).

## Warning: Removed 11 rows containing missing values (geom_point).
```

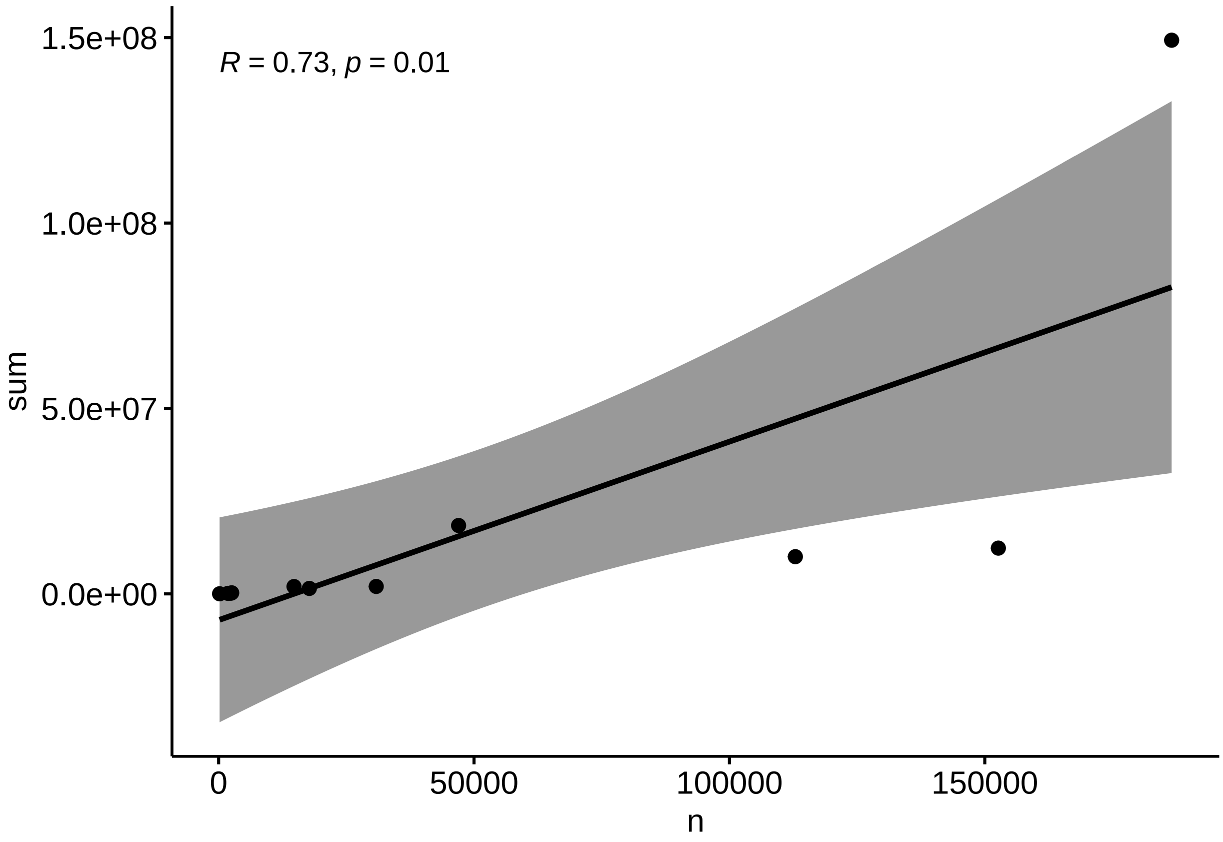


```r
ggscatter(my_data, x = "n", y = "BlastnSCO",
          add = "reg.line", conf.int = TRUE,
          cor.coef = TRUE, cor.method = "pearson")
```

```
## `geom_smooth()` using formula 'y ~ x'

## Warning: Removed 11 rows containing non-finite values (stat_smooth).

## Warning: Removed 11 rows containing non-finite values (stat_cor).

## Warning: Removed 11 rows containing missing values (geom_point).
```

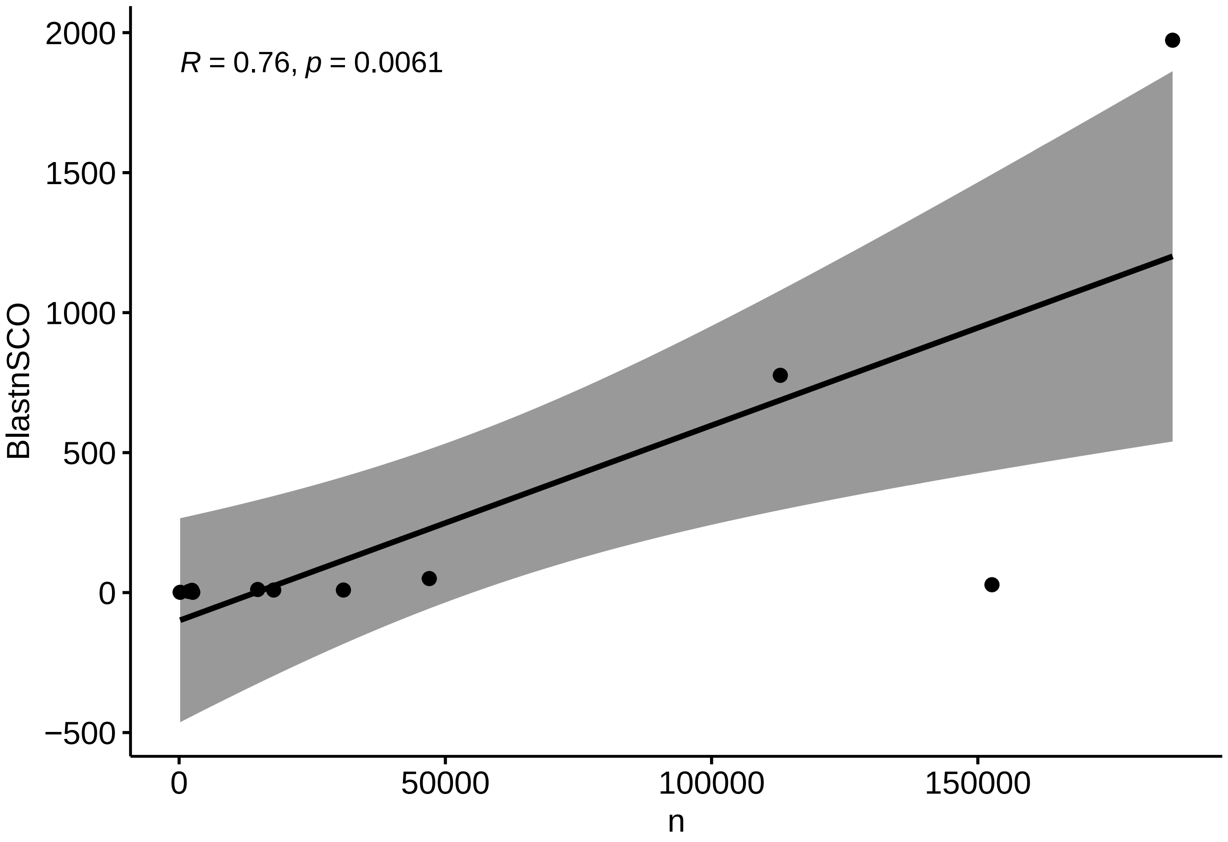


```r
ggscatter(my_data, x = "n", y = "BlastpSCO",
          add = "reg.line", conf.int = TRUE,
          cor.coef = TRUE, cor.method = "pearson")
```

```
## `geom_smooth()` using formula 'y ~ x'

## Warning: Removed 11 rows containing non-finite values (stat_smooth).

## Warning: Removed 11 rows containing non-finite values (stat_cor).

## Warning: Removed 11 rows containing missing values (geom_point).
```

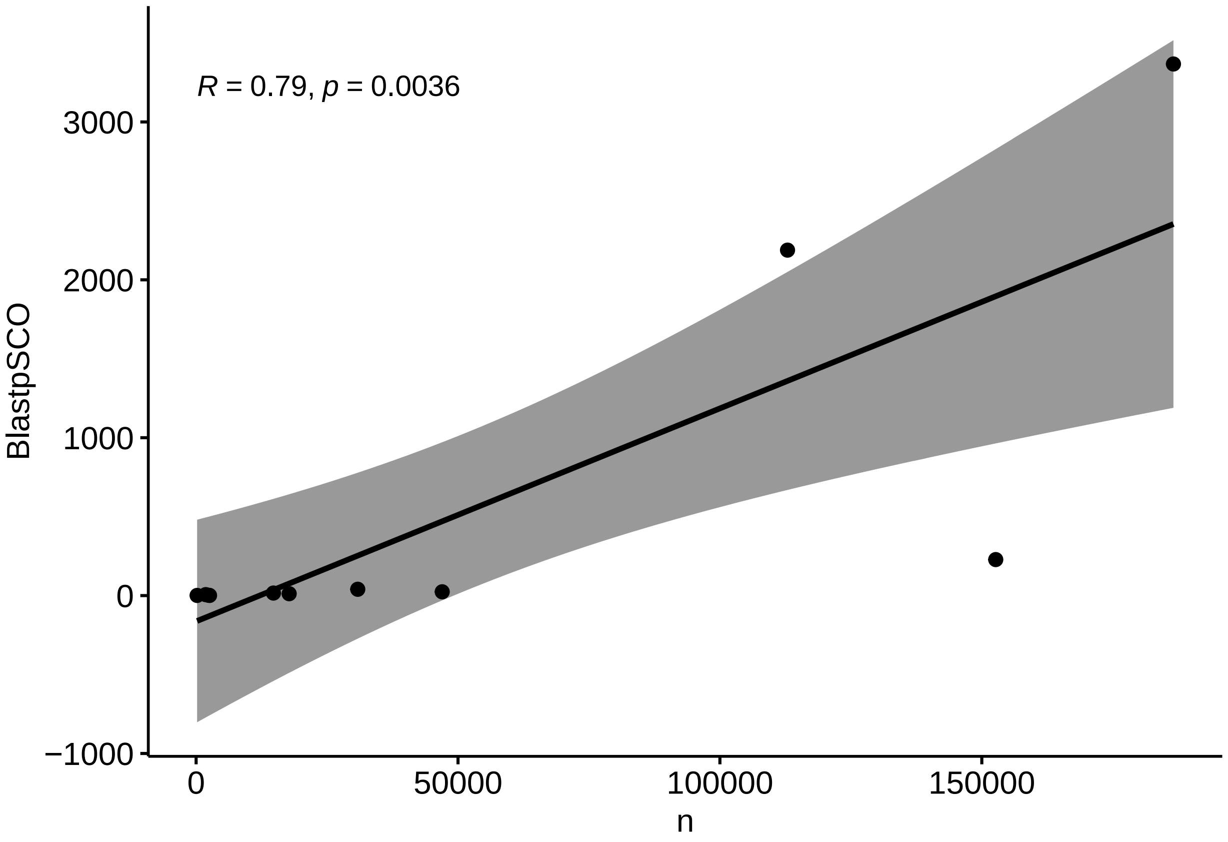


```r
ggscatter(my_data, x = "N50", y = "sum",
          add = "reg.line", conf.int = TRUE,
          cor.coef = TRUE, cor.method = "pearson")
```

```
## `geom_smooth()` using formula 'y ~ x'

## Warning: Removed 11 rows containing non-finite values (stat_smooth).

## Warning: Removed 11 rows containing non-finite values (stat_cor).

## Warning: Removed 11 rows containing missing values (geom_point).
```

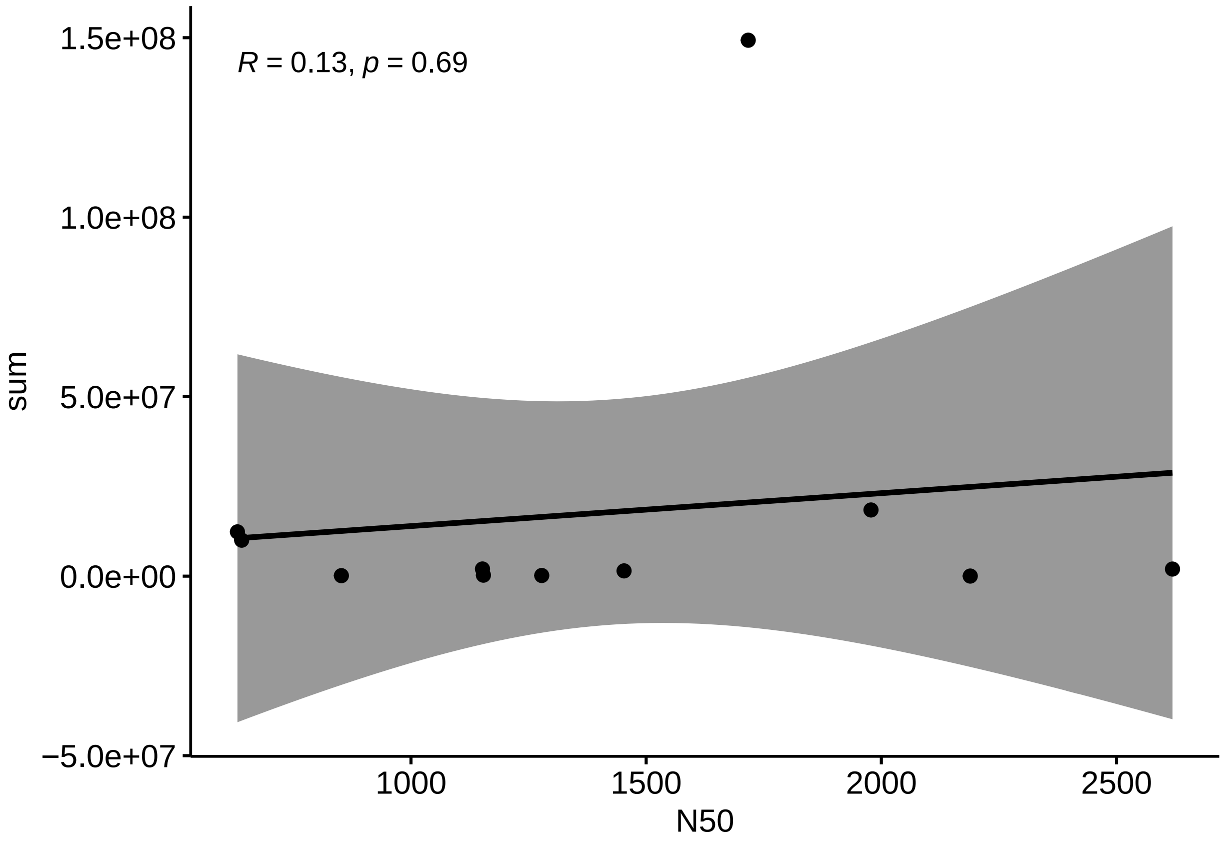


```
ggscatter(my_data, x = "N50", y = "BlastnSCO",
          add = "reg.line", conf.int = TRUE,
          cor.coef = TRUE, cor.method = "pearson")
```

```
## `geom_smooth()` using formula 'y ~ x'

## Warning: Removed 11 rows containing non-finite values (stat_smooth).

## Warning: Removed 11 rows containing non-finite values (stat_cor).

## Warning: Removed 11 rows containing missing values (geom_point).
```

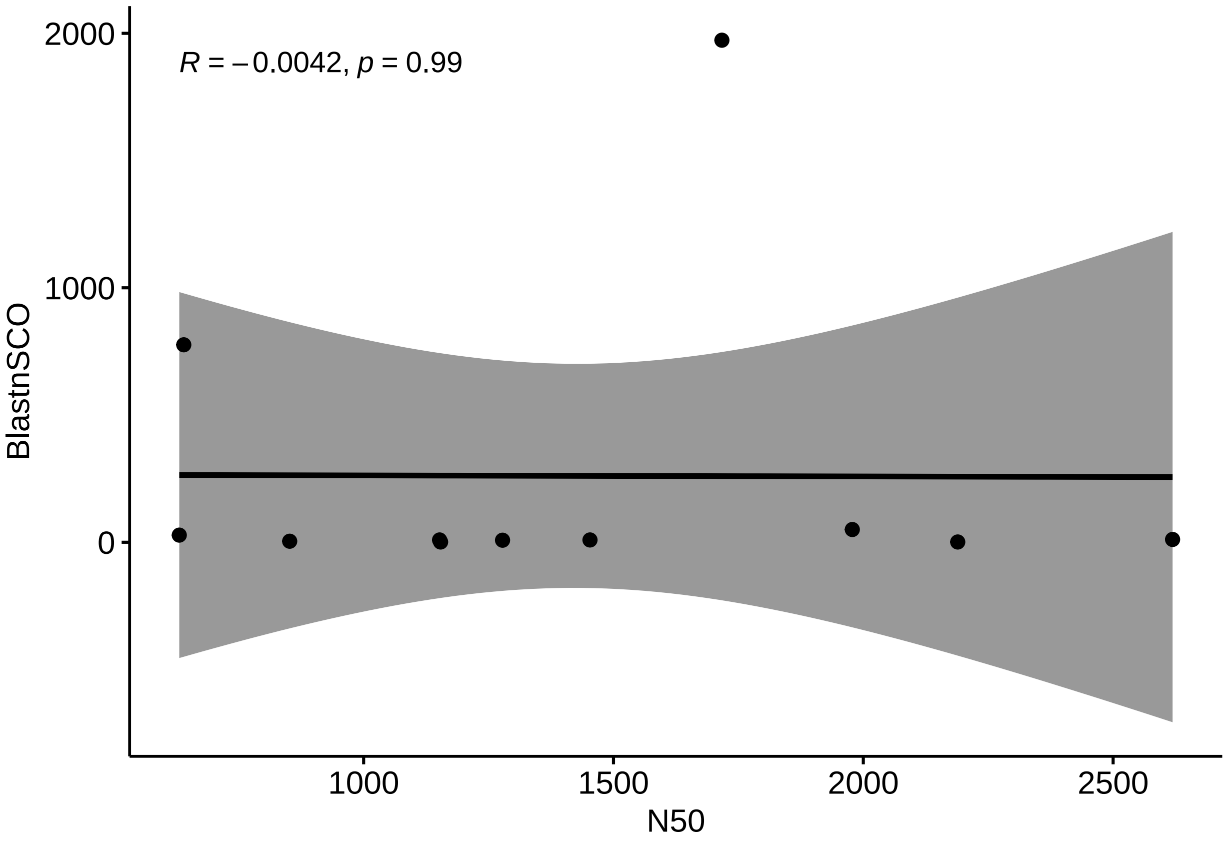


```r
ggscatter(my_data, x = "N50", y = "BlastpSCO",
          add = "reg.line", conf.int = TRUE,
          cor.coef = TRUE, cor.method = "pearson")
```

```
## `geom_smooth()` using formula 'y ~ x'
```

```
## Warning: Removed 11 rows containing non-finite values (stat_smooth).
```

```
## Warning: Removed 11 rows containing non-finite values (stat_cor).
```

```
## Warning: Removed 11 rows containing missing values (geom_point).
```

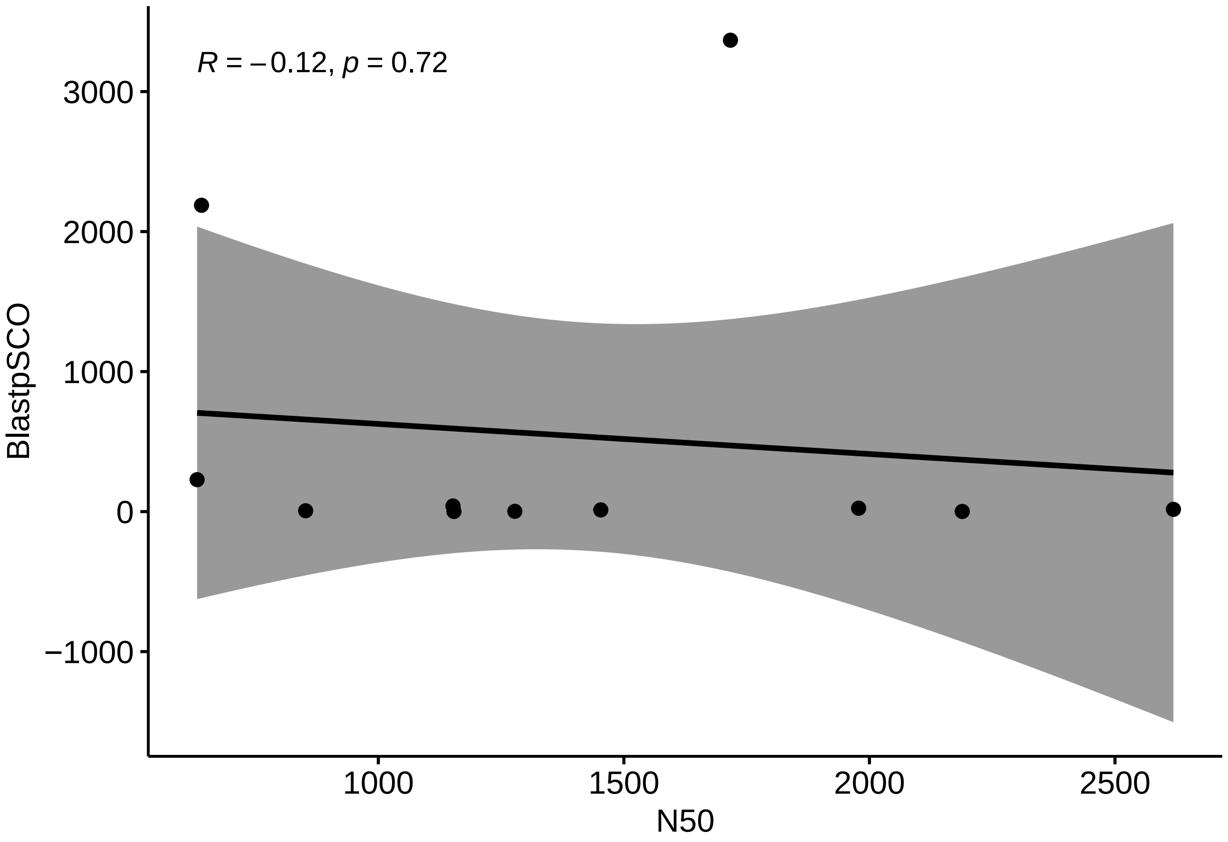


```r
ggscatter(my_data, x = "sum", y = "BlastnSCO",
          add = "reg.line", conf.int = TRUE,
          cor.coef = TRUE, cor.method = "pearson")
```

```
## `geom_smooth()` using formula 'y ~ x'

## Warning: Removed 11 rows containing non-finite values (stat_smooth).

## Warning: Removed 11 rows containing non-finite values (stat_cor).

## Warning: Removed 11 rows containing missing values (geom_point).
```

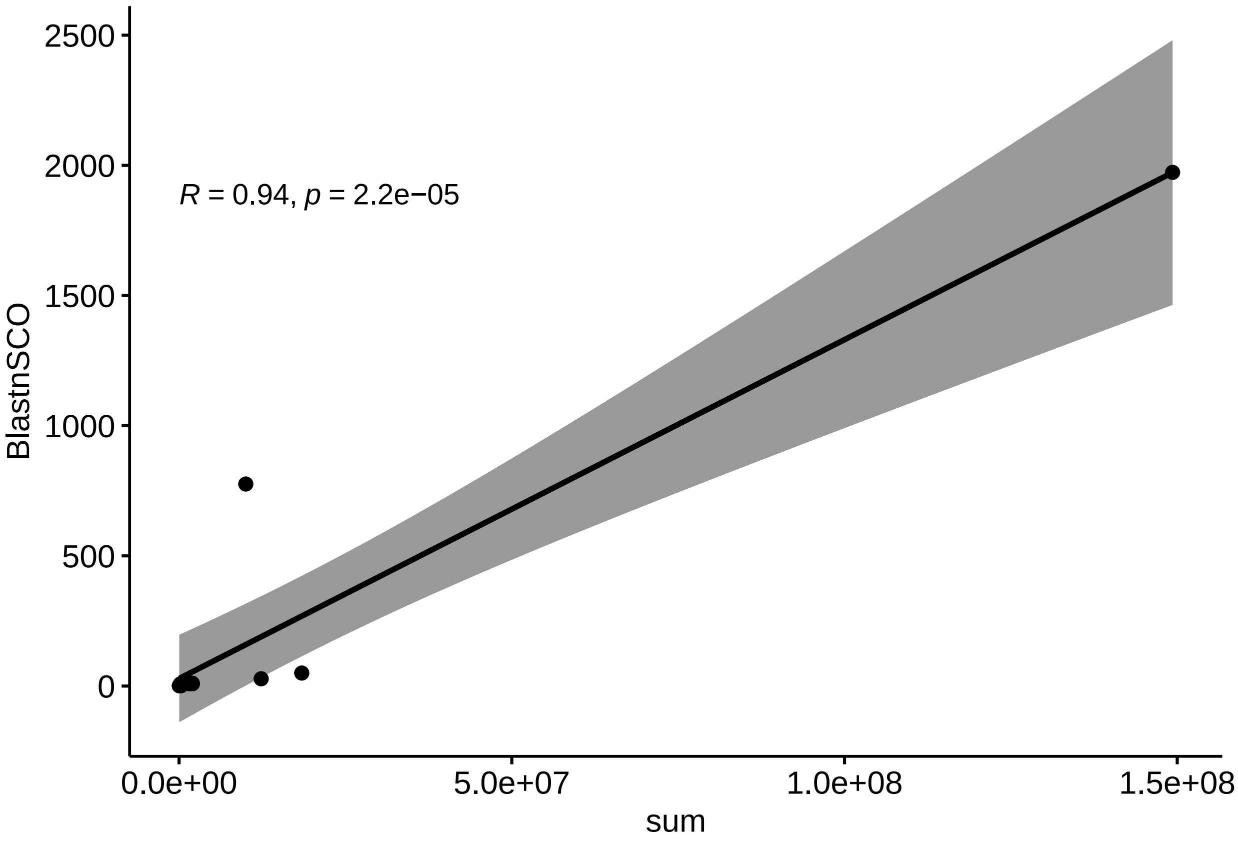


```r
ggscatter(my_data, x = "sum", y = "BlastpSCO",
          add = "reg.line", conf.int = TRUE,
          cor.coef = TRUE, cor.method = "pearson")
```

```
## `geom_smooth()` using formula 'y ~ x'

## Warning: Removed 11 rows containing non-finite values (stat_smooth).

## Warning: Removed 11 rows containing non-finite values (stat_cor).

## Warning: Removed 11 rows containing missing values (geom_point).
```

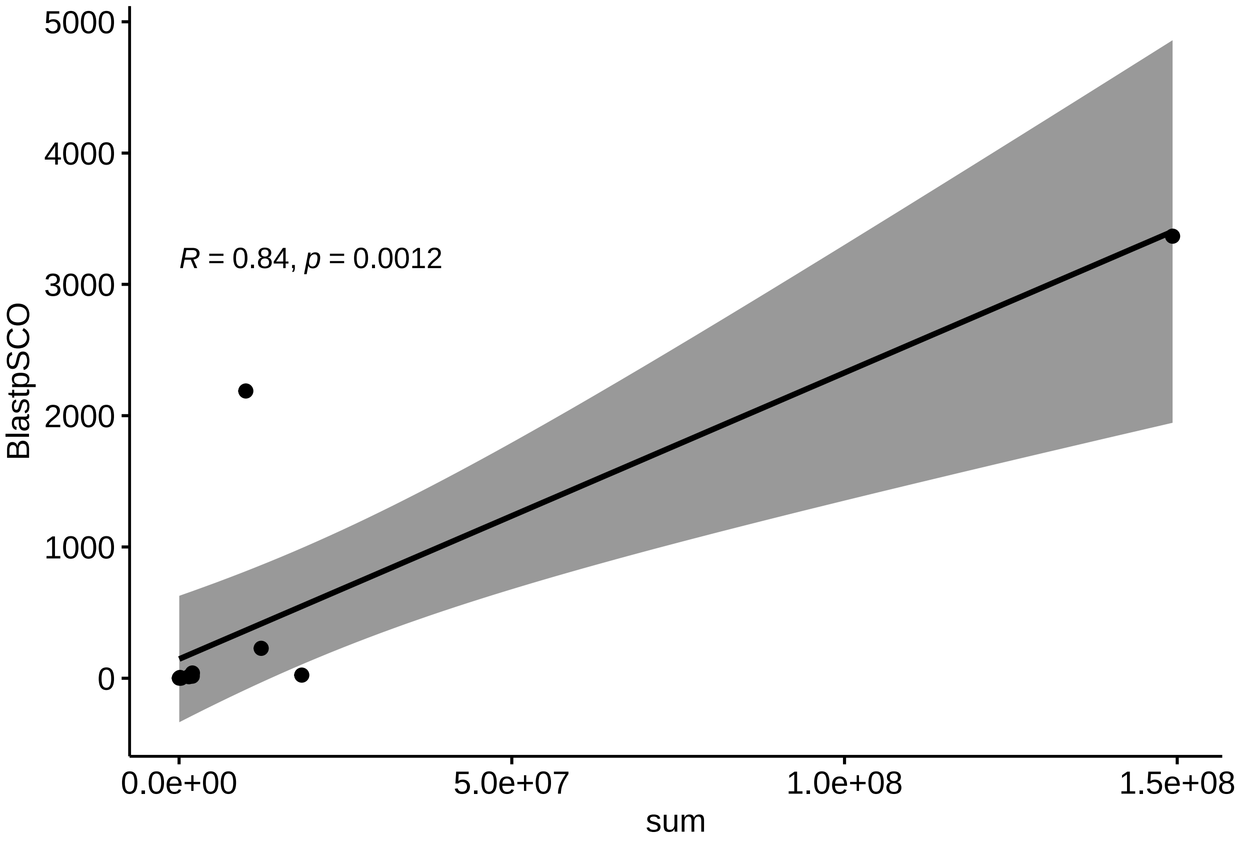


```r
ggscatter(my_data, x = "BlastnSCO", y = "BlastpSCO",
          add = "reg.line", conf.int = TRUE,
          cor.coef = TRUE, cor.method = "pearson")
```

```
## `geom_smooth()` using formula 'y ~ x'

## Warning: Removed 11 rows containing non-finite values (stat_smooth).

## Warning: Removed 11 rows containing non-finite values (stat_cor).

## Warning: Removed 11 rows containing missing values (geom_point).
```

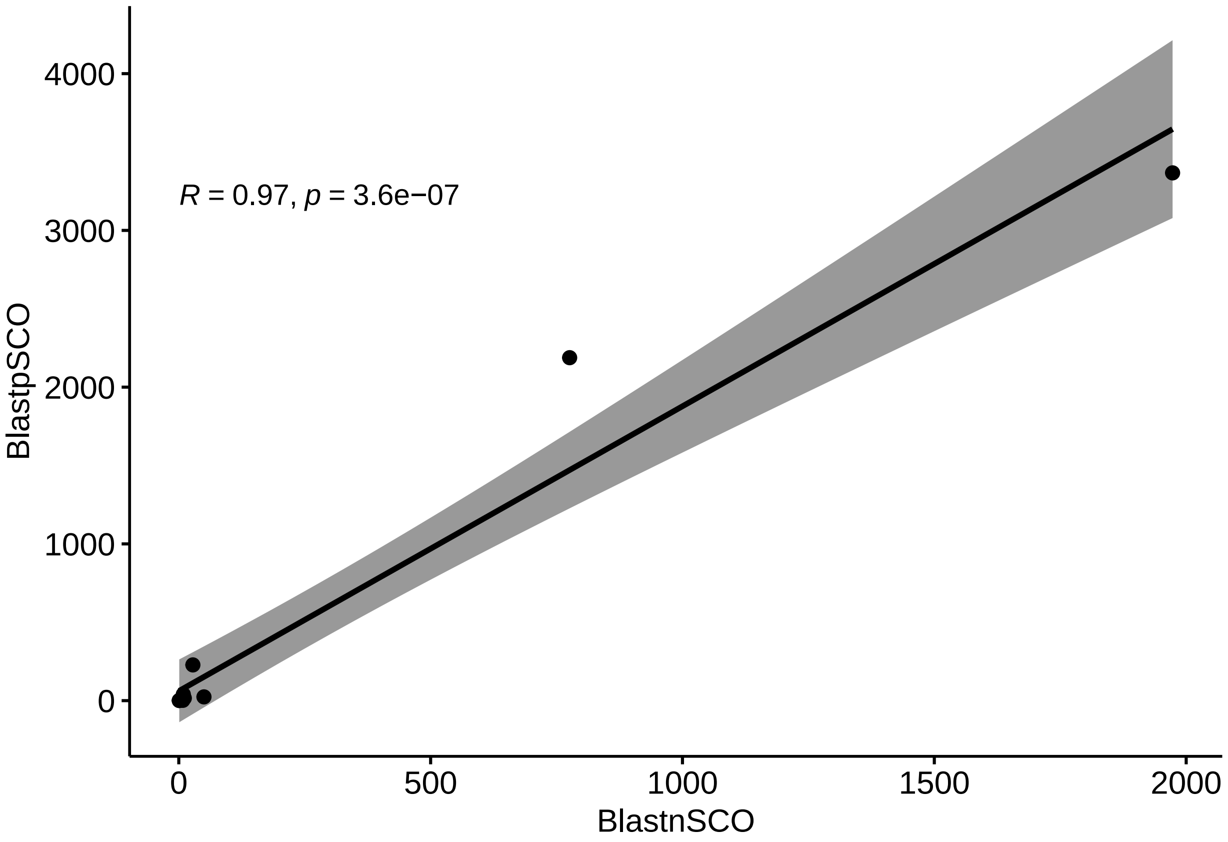
R = 0.97, p = 3.6e−07
BlastpSCO
4000
3000
2000
1000
0
0
500
1000
1500
2000
BlastnSCO
